# Supplementary material for: Antifreeze protein dispersion in eelpouts and related fishes reveals migration and climate alteration within the last 20 Ma
Source: PLoS One. 2020 Dec 15;15(12):e0243273. doi: 10.1371/journal.pone.0243273 (PMC7737890; doi:10.1371/journal.pone.0243273)
Supplement: S1 Fig — GenBank accession #s, sequentially top to bottom as in the alignment, are KC016052, KJ205263, KC517318, HQ712639, HQ713113, HQ713057, KJ205118, KC016016, JQ685890, KC015305, EU752057, EF427917. Since a COI sequence was not available for the Antarctic eelpout, Lycodichthys dearborni, the one for Lycodichthys antarcticus was used instead. Asterisks under the alignment indicate perfect conservation of a base. (DOCX) [file pone.0243273.s001.docx]

ocean pout, *Zoarces americanus* CCTTTATCTAGTATTTGGTGCCTGAGCCGGAATAGTTGGCACAGCCCTAAGCCTCCTCAT

viviparous eelpout, *Zoarces viviparus* CCTTTATCTAGTATTTGGTGCCTGGGCCGGAATAGTTGGCACAGCCCTAAGCCTCCTCAT

notched-fin eelpout, *Zoarces elongatus* ------------------------------------------------------------

Canadian eelpout, *Lycodes polaris* CCTTTATCTAGTATTTGGTGCCTGAGCCGGAATAGTTGGCACAGCTCTAAGCCTACTCAT

*Pachycara brachycephalum* CCTTTATCTAGTATTTGGTGCCTGGGCCGGAATAGTGGGCACAGCTCTAAGCCTCCTCAT

*Lycodichthys antarcticus* CCTTTATCTAGTATTTGGTGCCTGAGCCGGAATAGTGGGTACAGCTCTAAGCCTCCTCAT

rock gunnel, *Pholis gunnellus* CCTTTATCTAGTATTTGGTGCATGAGCCGGAATAGTAGGCACAGCTCTAAGCCTCCTCAT

radiated shanny, *Ulvaria subbifurcata* CCTTTATCTAGTATTTGGTGCATGAGCCGGAATAGTAGGCACAGCTCTAAGCCTCCTTAT

Alaskan ronquil, *Bathymaster caeruleofasciatus* CCTTTATCTAGTATTTGGTGCCTGAGCCGGAATGGTAGGTACAGCTCTAAGCCTCCTCAT

wrymouth, *Cryptacanthodes maculatus* CCTTTATCTAGTATTTGGTGCATGAGCCGGAATAGTAGGCACAGCTCTAAGCCTCCTCAT

Atlantic wolffish, *Anarhichas lupus* CCTTTATCTAGTATTTGGTGCATGAGCCGGAATGGTAGGCACAGCCCTAAGCCTCCTCAT

spotted wolffish, *Anarhichas minor* CCTTTATCTAGTATTTGGTGCATGAGCCGGAATGGTAGGCACAGCCCTAAGCCTCCTCAT

ocean pout, *Zoarces americanus* TCGAGCGGAGCTAAGCCAACCCGGCGCCCTCTTGGGGGATGACCAAATCTACAATGTCAT

viviparous eelpout, *Zoarces viviparus* TCGAGCGGAGCTAAGCCAGCCCGGCGCCCTCTTGGGGGACGACCAAATTTATAATGTCAT

notched-fin eelpout, *Zoarces elongatus* ----------CTAAGCCAGCCCGGCGCCCTCTTGGGGGACGACCAAATTTATAATGTTAT

Canadian eelpout, *Lycodes polaris* TCGAGCGGAGCTAAGCCAACCCGGCGCCCTCCTGGGGGACGACCAAATTTACAATGTCCT

*Pachycara brachycephalum* TCGAGCGGAGCTAAGCCAACCCGGCGCCCTCCTGGGGGACGACCAAATTTATAATGTCCT

*Lycodichthys antarcticus* TCGAGCGGAGCTAAGCCAACCCGGCGCCCTCCTGGGGGACGACCAAATTTACAATGTCCT

rock gunnel, *Pholis gunnellus* TCGAGCAGAGTTAAGCCAGCCCGGCGCCCTACTAGGCGACGACCAAATTTATAATGTAAT

radiated shanny, *Ulvaria subbifurcata* TCGGGCGGAGTTAAGCCAACCCGGCGCCCTCTTAGGAGACGACCAAATTTATAACGTAAT

Alaskan ronquil, *Bathymaster caeruleofasciatus* TCGAGCAGAGCTAAGCCAACCCGGTGCCCTCTTAGGGGACGACCAAATTTATAATGTAAT

wrymouth, *Cryptacanthodes maculatus* TCGAGCAGAGCTAAGCCAGCCCGGCGCCCTTTTAGGAGACGACCAAATTTATAACGTAAT

Atlantic wolffish, *Anarhichas lupus* TCGAGCGGAGCTAAGCCAACCCGGCGCCCTCTTGGGGGACGACCAAATTTATAATGTGAT

spotted wolffish, *Anarhichas minor* TCGAGCGGAGCTAAGCCAACCCGGCGCCCTCTTGGGGGACGACCAAATTTATAATGTGAT

******* ***** ***** * ** ** ******** ** ** ** *

ocean pout, *Zoarces americanus* TGTTACAGCACATGCGTTCGTAATAATCTTCTTTATAGTAATACCAATCATGATTGGAGG

viviparous eelpout, *Zoarces viviparus* TGTTACAGCACATGCGTTCGTAATAATCTTCTTTATAGTAATACCAATCATGATCGGAGG

notched-fin eelpout, *Zoarces elongatus* TGTTACAGCACATGCGTTCGTAATAATCTTCTTTATAGTAATACCAATCATGATCGGAGG

Canadian eelpout, *Lycodes polaris* TGTTACAGCGCATGCGTTCGTAATAATTTTCTTTATAGTAATACCAATTATGATTGGGGG

*Pachycara brachycephalum* TGTTACAGCGCATGCGTTCGTAATAATTTTCTTTATAGTAATACCAATTATAATCGGGGG

*Lycodichthys antarcticus* TGTTACAGCACATGCGTTCGTAATAATCTTCTTTATAGTAATACCAATTATGATCGGGGG

rock gunnel, *Pholis gunnellus* TGTTACAGCACATGCATTCGTAATAATTTTCTTTATAGTAATACCAATCATGATCGGGGG

radiated shanny, *Ulvaria subbifurcata* TGTTACGGCACATGCGTTCGTAATAATTTTCTTTATAGTAATACCAATCATGATTGGAGG

Alaskan ronquil, *Bathymaster caeruleofasciatus* TGTTACAGCACATGCATTCGTAATAATTTTCTTTATAGTAATACCAATCATGATTGGAGG

wrymouth, *Cryptacanthodes maculatus* CGTTACAGCGCATGCGTTCGTAATAATTTTCTTTATAGTAATACCAATCATGATTGGGGG

Atlantic wolffish, *Anarhichas lupus* TGTTACGGCACATGCGTTCGTAATAATTTTCTTTATAGTGATACCAATTATAATTGGGGG

spotted wolffish, *Anarhichas minor* TGTTACGGCACATGCGTTCGTAATAATTTTCTTTATAGTGATACCAATTATAATTGGAGG

***** ** ***** *********** *********** ******** ** ** ** **

ocean pout, *Zoarces americanus* GTTTGGAAACTGGCTTGTCCCCTTAATGATCGGAGCCCCAGACATAGCATTTCCCCGAAT

viviparous eelpout, *Zoarces viviparus* GTTCGGAAACTGGCTTGTCCCCTTAATGATCGGGGCCCCAGATATAGCATTTCCCCGAAT

notched-fin eelpout, *Zoarces elongatus* GTTCGGAAACTGGCTTGTCCCCTTAATGATCGGGGCCCCAGATATAGCATTTCCCCGAAT

Canadian eelpout, *Lycodes polaris* TTTTGGAAACTGGCTCGTGCCCCTAATAATCGGGGCCCCGGACATGGCATTCCCCCGAAT

*Pachycara brachycephalum* GTTTGGAAACTGGCTTGTACCCTTGATAATCGGGGCCCCGGACATAGCATTTCCCCGAAT

*Lycodichthys antarcticus* CTTTGGAAACTGACTTGTGCCCTTAATAATCGGGGCCCCGGACATAGCATTTCCCCGAAT

rock gunnel, *Pholis gunnellus* CTTCGGAAACTGACTCATCCCCTTAATGATTGGGGCCCCAGACATGGCGTTCCCCCGTAT

radiated shanny, *Ulvaria subbifurcata* GTTTGGAAACTGACTCATTCCCTTAATGATTGGAGCCCCAGACATGGCGTTTCCTCGAAT

Alaskan ronquil, *Bathymaster caeruleofasciatus* CTTTGGAAACTGACTTATTCCCCTAATGATCGGGGCTCCAGACATAGCATTTCCCCGAAT

wrymouth, *Cryptacanthodes maculatus* TTTCGGAAACTGGCTTGTTCCCTTAATGATTGGAGCTCCAGACATGGCATTTCCCCGAAT

Atlantic wolffish, *Anarhichas lupus* TTTCGGAAACTGGCTTGTCCCCCTAATGATTGGGGCCCCGGACATAGCATTTCCCCGGAT

spotted wolffish, *Anarhichas minor* TTTCGGAAACTGGCTTGTCCCCCTAATGATTGGGGCCCCAGACATAGCATTTCCCCGGAT

** ******** ** * *** * ** ** ** ** ** ** ** ** ** ** ** **

ocean pout, *Zoarces americanus* AAATAACATGAGCTTTTGACTCCTTCCTCCATCTTTTCTTCTCCTCCTTGCCTCCTCAGG

viviparous eelpout, *Zoarces viviparus* AAACAACATAAGCTTTTGACTCCTCCCTCCATCTTTTCTTCTCCTCCTTGCCTCCTCGGG

notched-fin eelpout, *Zoarces elongatus* AAACAACATAAGCTTTTGACTCCTCCCTCCATCTTTTCTTCTCCTCCTTGCCTCCTCGGG

Canadian eelpout, *Lycodes polaris* AAACAACATGAGCTTTTGACTCCTTCCCCCATCTTTTCTTCTCCTCCTTGCCTCTTCGGG

*Pachycara brachycephalum* AAACAACATGAGCTTTTGACTCCTTCCCCCATCTTTCCTCCTCCTCCTTGCTTCTTCGGG

*Lycodichthys antarcticus* AAACAACATGAGCTTTTGACTCCTTCCCCCCTCTTTTCTCCTCCTCCTTGCCTCTTCGGG

rock gunnel, *Pholis gunnellus* AAATAACATGAGTTTTTGGCTTCTCCCTCCTTCTTTCCTTCTCCTCCTTGCCTCTTCTGG

radiated shanny, *Ulvaria subbifurcata* AAATAATATGAGTTTTTGACTTCTTCCTCCCGCCTTCCTGCTCCTCCTTGCCTCTTCTGG

Alaskan ronquil, *Bathymaster caeruleofasciatus* AAACAATATGAGCTTTTGACTTCTTCCTCCCTCTTTCCTTCTCCTTCTTGCCTCCTCAGG

wrymouth, *Cryptacanthodes maculatus* AAACAACATGAGTTTTTGACTCCTTCCCCCCTCTTTCCTTCTTCTCCTTGCCTCTTCTGG

Atlantic wolffish, *Anarhichas lupus* AAACAACATGAGCTTTTGACTTCTACCCCCATCTTTTCTCCTTCTCCTTGCCTCTTCCGG

spotted wolffish, *Anarhichas minor* AAACAAAATGAGCTTTTGACTTCTCCCCCCATCTTTTCTCCTTCTCCTTGCCTCTTCCGG

*** ** ** ** ***** ** ** ** ** * ** ** ** ** ***** ** ** **

ocean pout, *Zoarces americanus* GGTAGAGGCAGGAGCTGGCACGGGATGAACAGTCTACCCCCCTCTTTCTGGGAACTTGGC

viviparous eelpout, *Zoarces viviparus* GGTAGAAGCAGGAGCTGGCACGGGGTGAACAGTCTACCCCCCTCTTTCTGGAAACCTGGC

notched-fin eelpout, *Zoarces elongatus* GGTAGAGGCAGGAGCTGGCACGGGGTGAACAGTCTACCCTCCTCTTTCTGGAAACTTGGC

Canadian eelpout, *Lycodes polaris* GGTAGAGGCGGGTGCTGGGACAGGATGGACAGTTTACCCCCCTCTCTCTGGTAACTTAGC

*Pachycara brachycephalum* GGTAGAGGCGGGTGCTGGAACGGGGTGAACAGTTTACCCCCCTCTTTCTGGTAACTTAGC

*Lycodichthys antarcticus* GGTAGAGGCGGGTGCTGGGACAGGATGAACAGTGTACCCCCCTCTTTCTGGTAACTTAGC

rock gunnel, *Pholis gunnellus* GGTTGAGGCGGGAGCTGGTACAGGGTGAACGGTATACCCGCCCCTTTCTGGTAATTTAGC

radiated shanny, *Ulvaria subbifurcata* GGTAGAGGCGGGCGCCGGAACAGGGTGAACAGTTTACCCGCCCCTTTCGGGTAATTTAGC

Alaskan ronquil, *Bathymaster caeruleofasciatus* TGTAGAGGCGGGGGCCGGAACAGGATGAACAGTTTATCCCCCTCTTGCTGGCAATTTAGC

wrymouth, *Cryptacanthodes maculatus* CGTAGAGGCGGGGGCCGGGACAGGATGAACAGTTTATCCGCCCCTTTCTGGTAACTTAGC

Atlantic wolffish, *Anarhichas lupus* GGTAGAGGCTGGAGCTGGGACAGGGTGAACAGTTTATCCCCCGCTCTCGGGTAACTTAGC

spotted wolffish, *Anarhichas minor* GGTAGAGGCTGGAGCTGGGACAGGGTGAACAGTTTATCCCCCTCTCTCGGGTAACTTAGC

** ** ** ** ** ** ** ** ** ** ** ** ** ** ** * ** ** * **

ocean pout, *Zoarces americanus* CCATGCGGGAGCTTCTGTTGATTTAACAATCTTCTCCCTTCACTTAGCGGGGATCTCTTC

viviparous eelpout, *Zoarces viviparus* CCATGCGGGGGCTTCTGTTGATTTAACAATCTTTTCCCTTCACCTAGCGGGAATCTCTTC

notched-fin eelpout, *Zoarces elongatus* CCATGCGGGGGCTTCTGTTGATTTAACAATCTTTTCCCTCCACCTAGCGGGAATCTCTTC

Canadian eelpout, *Lycodes polaris* CCATGCAGGGGCCTCCGTTGATTTAACAATTTTCTCTCTCCACCTAGCAGGAATCTCTTC

*Pachycara brachycephalum* CCACGCAGGGGCCTCCGTTGATTTAACAATCTTCTCCCTTCACTTAGCAGGGATTTCTTC

*Lycodichthys antarcticus* CCACGCAGGGGCCTCCGTTGATTTAACAATCTTCTCCCTTCACTTAGCAGGGATTTCTTC

rock gunnel, *Pholis gunnellus* GCACGCCGGGGCCTCTGTTGATTTAACAATCTTTTCTCTTCATCTGGCGGGGATTTCTTC

radiated shanny, *Ulvaria subbifurcata* CCATGCCGGAGCCTCTGTTGATTTAACAATCTTTTCTCTCCACTTAGCAGGAATTTCTTC

Alaskan ronquil, *Bathymaster caeruleofasciatus* CCATGCAGGAGCTTCTGTTGATTTAACAATCTTTTCTCTTCATTTAGCAGGAATTTCTTC

wrymouth, *Cryptacanthodes maculatus* CCATGCAGGAGCCTCTGTTGATTTAACAATCTTTTCTCTTCATTTAGCAGGGATTTCTTC

Atlantic wolffish, *Anarhichas lupus* CCATGCAGGAGCCTCTGTTGATTTGACAATCTTTTCCCTTCACCTAGCAGGAATTTCTTC

spotted wolffish, *Anarhichas minor* CAATGCAGGAGCCTCTGTTGATTTAACAATCTTTTCCCTTCACTTAGCAGGAATTTCTTC

* ** ** ** ** ******** ***** ** ** ** ** * ** ** ** *****

ocean pout, *Zoarces americanus* GATCCTCGGAGCAATTAACTTCATTACAACCATCATTAACATAAAACCCCCTGCGATTTC

viviparous eelpout, *Zoarces viviparus* GATCCTCGGAGCAATTAATTTCATTACAACCATCATTAACATGAAGCCCCCTGCGATTTC

notched-fin eelpout, *Zoarces elongatus* GATCCTCGGGGCAATTAATTTCATTACAACCATCATTAACATGAAGCCCCCTGCGATTTC

Canadian eelpout, *Lycodes polaris* AATCCTCGGGGCAATTAATTTTATTACGACCATCATTAACATGAAGCCCCCTGCGATCTC

*Pachycara brachycephalum* GATCCTCGGGGCAATTAACTTCATTACAACCATCATTAACATGAAGCCCCCTGCGATCTC

*Lycodichthys antarcticus* GATCCTCGGGGCAATTAATTTCATTACAACCATCATTAACATGAAGCCCCCTGCGATCTC

rock gunnel, *Pholis gunnellus* AATTCTTGGGGCAATTAACTTTATCACAACCATTATTAACATAAAACCTCCTGCCATTTC

radiated shanny, *Ulvaria subbifurcata* AATTCTAGGGGCAATCAACTTCATTACGACTATTATTAACATGAAGCCCCCCGCCATTTC

Alaskan ronquil, *Bathymaster caeruleofasciatus* AATCCTTGGAGCAATTAACTTCATTACAACCATTATTAACATGAAACCGCCTGCCATTTC

wrymouth, *Cryptacanthodes maculatus* AATCCTAGGGGCAATCAACTTCATTACAACTATTATTAACATGAAACCCCCTGCCATTTC

Atlantic wolffish, *Anarhichas lupus* AATCCTTGGGGCAATTAATTTCATTACAACCATTATTAACATGAAACCCCCAGCGATTTC

spotted wolffish, *Anarhichas minor* AATCCTTGGGGCAATTAATTTCATTACAACCATTATTAACATGAAACCCCCTGCGATTTC

** ** ** ***** ** ** ** ** ** ** ******** ** ** ** ** ** **

ocean pout, *Zoarces americanus* TCAGTACCAAACACCCCTCTTCGTCTGATCTGTCCTTATCACGGCTGTCCTACTACTCCT

viviparous eelpout, *Zoarces viviparus* TCAGTACCAGACACCCCTCTTCGTCTGATCTGTCCTTATTACGGCTGTCCTACTACTCCT

notched-fin eelpout, *Zoarces elongatus* TCAGTACCAGACACCCCTCTTCGTCTGATCTGTCCTTATCACGGCTGTCCTACTACTCCT

Canadian eelpout, *Lycodes polaris* TCAGTACCAGACGCCCCTCTTCGTCTGGTCCGTGCTCATTACGGCGGTCCTACTGCTCCT

*Pachycara brachycephalum* TCAGTACCAGACACCCCTCTTCGTCTGATCAGTACTTATCACGGCGGTCCTGCTCCTCCT

*Lycodichthys antarcticus* TCAGTACCAGACACCCCTCTTCGTCTGATCGGTACTTATCACGGCGGTCCTGCTCCTCCT

rock gunnel, *Pholis gunnellus* TCAGTATCAAACACCGCTCTTCGTTTGATCCGTACTTATTACCGCCGTTCTTCTGCTCCT

radiated shanny, *Ulvaria subbifurcata* TCAATATCAAACACCACTATTCGTTTGATCAGTACTTATTACAGCCGTCCTCCTACTCCT

Alaskan ronquil, *Bathymaster caeruleofasciatus* TCAATATCAAACACCCCTTTTCGTTTGATCAGTACTCATTACCGCAGTCCTTCTACTCCT

wrymouth, *Cryptacanthodes maculatus* TCAGTACCAAACACCCCTCTTCGTTTGATCAGTGCTTATTACCGCCGTCCTCCTACTCCT

Atlantic wolffish, *Anarhichas lupus* TCAGTACCAAACACCCCTCTTCGTTTGATCAGTGCTTATTACGGCCGTCCTGCTTCTCCT

spotted wolffish, *Anarhichas minor* TCAGTACCAAACACCCCTCTTCGTTTGATCAGTGCTTATTACGGCCGTCCTGCTTCTCCT

*** ** ** ** ** ** ***** ** ** ** ** ** ** ** ** ** ** *****

ocean pout, *Zoarces americanus* CTCCCTCCCCGTGCTCGCAGCCGGTATCACAATGCTCCTAACAGACCGCAATCTCAACAC

viviparous eelpout, *Zoarces viviparus* CTCCCTCCCCGTGCTCGCAGCCGGTATTACAATGCTCCTAACAGACCGCAACCTCAACAC

notched-fin eelpout, *Zoarces elongatus* CTCCCTCCCCGTGCTCGCAGCCGGTATTACAATGCTCCTAACAGACCGCAACCTCAACAC

Canadian eelpout, *Lycodes polaris* CTCTCTCCCCGTCCTTGCAGCCGGAATTACCATGCTCCTGACAGATCGAAACCTTAATAC

*Pachycara brachycephalum* TTCTCTCCCCGTCCTCGCAGCTGGTATCACCATGCTCCTGACCGATCGTAACCTTAACAC

*Lycodichthys antarcticus* TTCTCTCCCGGTCCTCGCAGCTGGTATCACCATGCTCCTGACAGATCGTAACCTTAACAC

rock gunnel, *Pholis gunnellus* TTCCCTCCCCGTGCTAGCAGCCGGAATCACCATACTTCTGACAGATCGTAATCTTAACAC

radiated shanny, *Ulvaria subbifurcata* TTCTCTCCCTGTGCTTGCAGCCGGTATCACAATACTTCTGACCGACCGTAATCTCAATAC

Alaskan ronquil, *Bathymaster caeruleofasciatus* TTCTCTTCCCGTTCTTGCAGCTGGTATTACGATACTACTAACAGACCGTAATCTTAACAC

wrymouth, *Cryptacanthodes maculatus* CTCCCTTCCTGTACTTGCAGCTGGTATTACAATACTCTTAACAGATCGCAATCTTAATAC

Atlantic wolffish, *Anarhichas lupus* TTCCCTCCCCGTACTTGCAGCTGGTATTACCATACTCCTAACAGACCGTAACCTTAACAC

spotted wolffish, *Anarhichas minor* TTCTCTCCCCGTACTTGCAGCTGGTATTACTATACTCCTAACAGACCGTAACCTTAACAC

** ** ** ** ** ***** ** ** ** ** ** * ** ** ** ** ** ** **

ocean pout, *Zoarces americanus* CACCTTCTTCGACCCCGCCGGGGGAGGGGACCCAATCCTTTACCAACACTTG

viviparous eelpout, *Zoarces viviparus* CACCTTCTTTGACCCAGCCGGGGGAGGGGACCCAATCCTATACCAACACTTT

notched-fin eelpout, *Zoarces elongatus* CACCTTCTTTGACCCCGCCGGGGGAGGAGACCCAATCCTCTACCAACATTTG

Canadian eelpout, *Lycodes polaris* CACCTTCTTCGACCCCGCCGGAGGAGGAGACCCCATCCTTTATCAACATCTG

*Pachycara brachycephalum* CACCTTCTTCGACCCCGCCGGGGGAGGAGACCCAATCCTCTACCAACACCTA

*Lycodichthys antarcticus* CACCTTCTTCGACCCCGCCGGGGGAGGAGACCCAATCCTTTACCAGCACCTA

rock gunnel, *Pholis gunnellus* TACCTTTTTTGACCCCGCAGGAGGGGGTGACCCAATTCTTTACCAACACTTA

radiated shanny, *Ulvaria subbifurcata* CACCTTCTTCGACCCCGCCGGTGGAGGTGACCCAATTCTTTACCAACATTTA

Alaskan ronquil, *Bathymaster caeruleofasciatus* CACTTTCTTCGACCCTGCCGGGGGAGGAGA----------------------

wrymouth, *Cryptacanthodes maculatus* CACATTCTTCGACCCCGCTGGAGGAGGTGACCCAATCCTCTATCAACACTTG

Atlantic wolffish, *Anarhichas lupus* CACCTTCTTCGACCCCGCCGGAGGGGGGGACCCAATCCTTTATCAACACTTG

spotted wolffish, *Anarhichas minor* CACCTTCTTCGACCCCGCCGGAGGGGGGGACCCAATCCTTTATCAACACTTG

** ** ** ***** ** ** ** ** **
