# Supplementary material for: Antifreeze protein dispersion in eelpouts and related fishes reveals migration and climate alteration within the last 20 Ma
Source: PLoS One. 2020 Dec 15;15(12):e0243273. doi: 10.1371/journal.pone.0243273 (PMC7737890; doi:10.1371/journal.pone.0243273)
Supplement: S2 Fig — The maximum-likelihood tree was generated using the Kimura 2-parameter with invariant sites model and bootstrap values (%) are indicated at the nodes. The Alaskan ronquil was used as the outgroup and the scale bar represents an average of 0.02 changes per site. This tree was used to determine the placement of the radiated shanny, Antarctic eelpout and Pachycara brachycephalum relative to the other species in Fig 1. (DOCX) [file pone.0243273.s002.docx]

1 5 10 20 30 40 50 60 70

iiii i i i i i i i i ii i

**notched-fin eelpout-Q1**  mksviltgllfvllcvdhmssaNQ-ESVVAAVLIPINTALTVGMMTTRVVSPTGIPAEDIPRLISMQVNQAVPMGTTLMPDMVKFYCLCAPKN

**viviparous-eelpout-Q1†** mksviltgllfvllcvdhmssaNQ-ESVVAAVLIPINTALTVGMMTTRVVSPTGIPAEDIPRLVSMQVNQAVPMGTTLMPDMVKFYCLCAPKN

**ocean pout-Q1*** --------------cvdhmssaNQ-ESVVAATLIPINTALTVGMMTTRVVTPVGIPAEDIPRLVSMQVNQAVPMGTTLMPDMVKFYCLCAPKN

**ocean pout-Q2**  mksviltgllfvllcvdhmssaNQ-ESVVATQLIPINTALTLVMMTTRVIYPTGIPAEDIPRLVSMQVNQAVPMGTTLMPDMVKFYCLCAPKN

**Atlantic wolffish-Q1** mksailtgllfvllcvdhmssaHQ-ASVVATQLIPINTALTLVMMTTRVVFPTGIPAEDIPRLVSMQVNRAVPMGTTLMPDMVKFYCLCAPKTQRW

**Atlantic wolffish-Q2** ---ailtgllfvllcvdhmssaNQ-ASVVATQLIPINTALTLVMMTTAVVTPTGIPAEDIPRLVSMQVNRAVPMGTTLMPDMVKFYCLCAPKY

**Alaskan ronquil-Q1*** -----------------nmssaK-GDSVVATQLIPINTALTLVMMGTADVTPTGIPAEDIPRLVGMQVNRAVLMGTTLMPDMVKFYCLC

**ocean pout-Q3**  mksviltgllfvllcvdhmssaNQ-ESVVATQLIPINTALTLVMMTTRVIYPTGIPAEDIPRLVSMQVNQAVPMGTTLLPDMVKGYPL-T

**notched-fin eelpout-Q2** mksviltglffvllcvdhmssaNQ-ESVVAAVLIPINTALTVGMMTTRVVSPTGIPAEDIPRLISMQVNQVVPMGTTLMPDMVKGYAP-A

**viviparous eelpout-Q2†** mksviltglffvllcvnhmssaNQ-ESVVAAVLIPINTALTVGMMTTRVVSPTGIPAEDIPRLISMQVNQVVPMGTTLMPDMVKGYAP-A

**ocean pout-Q4*** --------------cvdhmssaNQ-ASVVATQLIPINTALTLVMMRSKVVTPMGIPAEDIPRLVSMQVNQAVACGTTLMPGMVKTYTP-AK

**viviparous eelpout-Q3†**  mksviltgllfvllcvdhmssaNQ-ASVVATQLIPINTALTLVMMKAKVVTPMGIPAEDIPRIVSMQVNQAVACGTTLMPGMVKTYTP-VK

**notched-fin eelpout-Q3** mksviltgllfvllcvdhmssaNQ-ASVVATQLIPINTALTLVMMKAKVATPMGIPAEDIPRIVSMQVNQAVACGTTLMPGMVKTYTP-VK

**ocean pout-Q5*** --------------cvdhmssaNQ-ASVVANQLIPINTALTLVMMRSEVVTPVGIPAEDIPRLVSMQVNRAVPLGTTLMPDMVKGYPP-A

**radiated shanny-Q1*** mnsviftglvfvllcvdnmssaASGQSVVANQLIPINTALTLVMMRAEVVSPLGIPAEDIPRLVSLQVNRAVPLGTTLTAEMVKGYSP-AK

**rock gunnel-Q1*** mnsailtgflfvllcvdnmtsaGSGKSVVANQLIPINTALTRIMMKAELVAPMGIPAEDIPRLVSLQVNRAVPMGTTLMPDMVKTYQP-AK

**viviparous eelpout-Q4†** mksviltgllfvllcvdhmssaNQ-ASVVANQLIPINTALTLVMMRSEVVTPMGIPAVDIPRLVSMQVNRAVPLGTTLMPDMVKGYTP-A

**notched-fin eelpout-Q4**  mksviltgllfvllcvdhmssaNQ-ASVVANQLIPINTALTLVMMRAEVVTPMGIPAVDIPRLVSMQVNRAVPLGTTLMPEMVKGYTP-A

**viviparous-eelpout-Q5†** mksviltgllfvllcvdhmssaDQ-ASVVANQLIPINTALTLVMMRAEVVTPMGIPAEDIPRLVSLQVNRAVPLGTTIMPDMVKGYAP-N

**Canadian eelpout-Q1** ----------------------NK-ASVVANQLIPINTALTLVMMRAEVVTPAGIPAEDIPRLVGLQVNRAVLIGTTLMPDMVKGYAP-Q

**spotted wolffish-Q1** mksailtgllfvllcvdhmssaHQ-ASIVANQLIPINTALTLIMMRAQVVTPLGIPAEDIPRIIGMQVNRAVALGTTLMPDMVKGYPP-N

**spotted wolffish-Q2** mksailtgllfvllcvdhmssaHQ-ASIVANQLIPINTALTPIMTKAQVVTPLGIPAEDIPRIIGMQVNRAVALGTTLMPDMVKGYPP-N

***P. brachycephalum*-1†** mksvvltgllfvllcvdhmssaNK-ASVVANQLIPINTALTLVMMKAEVVTPMGIPAEDIPRLIGMQVNRAVPLGTTLMPDMVKMYCLCI

**Antarctic eelpout-Q1**  mksvvltgllfvllcvdhmssaNK-ASVVANQLIPINTALTLIMMKAEVVTPMGIPAEDIPRIIGMQVNRAVPLGTTLMPDMVKNY---EK

**Antarctic eelpout-Q2** mksvvltgllfvllcvdhmssaNK-ASVVANQLIPINTALTLIMMKAEVVTPMGIPAEDIPKLMGMQVNRAVPLGTTLMPDMVKNY---EK

**Antarctic eelpout-Q3a** mksvvltgllfvllcvdhmssaNK-ASVVANQLIPINTALTLIMMKAEVVTPMGIPAEEIPNLVGMQVNRAVPLGTTLMPDMVKNY---ED

**Antarctic eelpout-Q4** mksvvltgllfvllcvdhmssaNK-ASVVANQLIPINTALTLVMMKAEVVTPMGIPAEEIPKLVGMQVNRAVPLGTTLMPDMVKNY---EK

**Antarctic eelpout-Q3b**  (tandemer) *GTTSPGLK*SVVANQLIPINTALTLVMMKAEEVSPKGIPSEEISKLVGMQVNRAVPLGTTLMPDMVKNY---EK

***P. brachycephalum*-Q2†** mksvvltgllfvllcvdhmssaNK--SVVANQLIPINTALTLVMMKAEEVSPKGIPAEEIPRLVGMQVNRAVPLGTTLMPDMVKNY---QK

**Antarctic eelpout-Q5b** (tandemer) *VTTCPGFK*SAVANQLIPINTALTLMMMKAEEVSPKGIPAEEIPKLVGMQVNRAVYLDQTLMPDMVKNY---ED

**Antarctic eelpout-Q6** --------------------------SVVANQLIPINTALTLVMMKAKEVSPKGIPAEEIPKLVGMQVNRAVYLDETLMPDMVKNY---EK

***P. brachycephalum*-Q3** ----------------------TK--SVVANQLIPINTALTLVMMKAEEVSPKGIPAEEIPRLVGMQVNRAVYLDETLMPDMVKNY---E

***P. brachycephalum*-Q4** mksvvltgllfvllcvdhmssaTK--SVVASQLIPINTALTPAMMKAKEVSPKGIPAEEMSKIVGMQVNRAVNLDETLMPDMVKTY---QK

**ocean pout-Q6†** --------------cvdhmssaNQ-ASVVATQLIPINTALTLVMMKAKVVTPMGIPAEEIPQIVGLQVDRAVPLGTTLMPDMVKTYCA-AK

**ocean pout-Q7** mksviltglflvllcvdhmssaNQ-ESVVATQLIPINTALTPIMMKGKVVTPMGIPFKEMSQIVGKQVNRAVPLGTTIMPEMVKGYAP-N

**Antarctic eelpout-sasB** ------------------------**GK**SVVA**KVK**IP**KG**T**V**LT**QD**M**LAV**K**AAE**PMGI**A**AED**LCKM**VGK**T**V**TED**V**EEDDSV**MP**E**MVKGY**CK**-**N**K**KC**

**wolf eel-sasB** ------------------------**GK**SVVA**KVK**IP**KG**T**V**LT**QD**M**LAV**K**AAE**PMGI**A**AED**LCK****M**VGK**T**V**TED**V**EEDDSI**MP**E**MVKGY**CK**-**N**K**KC**

**Antarctic eelpout-sasA** ------------------------**GK**S**L**VA**TVK**IP**KG**T**V**LT**QD**M**LTV**K**VAE**PMG**VA**AED**IFQM**VGK**T**V**TKD**V**EEDGSLL**P**EV**V**D**GY**CK**-**KRKC**

**wolf eel-sasA** ------------------------**GK**S**L**VA**KVK**IP**KG**T**V**LT**QD**M**LTV**K**VAE**PMGI**A**AED**IFQM**VGK**T**V**TKD**V**EEDDSLL**P**EV**V**D**GY**CK**-**KRKC**

**Atlantic wolffish-S1** mksailtgllfvllcvdhmssaSQ--SVVATQLIPINTALTPIMMKGKVVNPAGIPFAEMSQIVGKQVNRPVAKDETLMPNMVKTYRA-AK

**spotted wolffish-S1** mksailtgllfvllcvdhmssaSQ--SVVATQLIPINTALTPIMMKGKVVNPAGIPFAEMSQIVGKQVNRPVAKDETLMPNMVKTYRA-AK

**spotted wolffish-S2** mksailtgllfvllcvdhmssaSQ--SVVATQLIPINTALTPAMMKGKVVSPAGIPFAEMSQIVGKQVNRPVAKDETLMPNMVKTYRA-AK

**rock gunnel-S1*** mnsailtgflfvllcvdnmtsaGS--SVVASQLIPMNTALTPAMMKGVVVSPAGIPFAEMSRIVGKQVNQIVAKDQTLMPSMVKTYQP-AK

**radiated shanny-S1*** mnsailtgflfvllcv-nmssaAQ--SVVATQLIPINTALTPAMMKGMDVNPSGIPFTEKSTLVGKQVNQPVVKGQTLMRNMVKP

**ocean pout-S1** mksviltgllfvllcvdhm-taSQ--SVVATQLIPINTALTPAMMEGKVTNPIGIPFAEMSQIVGKQVNTPVAKGQTLMPNMVKTYVA-GK

**ocean pout-S2*** --------------cvdhi-taSQ--SVVATRLIPMNTALTPAMMEGKVTNPIGIPFAEMSQIVGKQVNRIVAKGQTLMPNMVKTYAA-GK

**ocean pout-S3** mksviltgllfvllcvdhm-taSQ--SVVATQLIPMNSALTPVMMEGKVTNPIGIPFAEMSQMVGKQVNRPVAKGQTIMPNMVKTYAA-GK

**ocean pout-S4*** --------------cvdhm-taSQ--SVVATQLIPMNTALTPVMMEGKVTNPIGIPFAEMSQIVGKQVNTPVAKGQTIMPNMVKTYAA-GK

**viviparous eelpout-S1** mkcviltgllfvllcvdhmssaGE--SVVATQLIPINTALTPAMMAGKVTNPSGIPFAEMSQIVGKQVNTPVAKGQTLMPDMVKTYVP-AK

**notched-fin eelpout-S1** mksviltgllfvllcvdhmssaGE--SVVATQLIPINTALTPAMMEGKVTNPSGIPFAEMSQIVGKQVNTPVAKGQTLMPGMVKTYVP-AK

**viviparous eelpout-S2** ----------------------GE--SVVATQLIPMNTALTLAMIEGKVTNPSGIPFAEKSQIVGKQVNTPVAKGQTLMPDMVKTYVP-AK

**viviparous eelpout-S3†** mkcviltgllfvllcvdhmssaGE--SVVATQLIPMNTALTPAMMAGKVTNPSGIPFAEMLQIVGKQVNVIVAKGQTIMPNMVKTYAA-GK

**viviparous eelpout-S4†** mkcviltgllfvllcvdhmssaGE--SVVATQLIPMNTALTLAMIEGKVTNPSGIPFAEKSQIVGKQVNVIVAKGQTIMPIMVKTYVP-AK

**viviparous eelpout-S5†** mksviltgllfvllcvdhmssaGQ--SVVATQLIPMNTALTPAMMEGKVTNPSGIPFAEMSQIVGKQVNVIVPKGHTIMPIMVKTYA--GK

**notched-fin eelpout-S2** mksviltgllfvllcvdhmssaGE--SVVATQLIPINTALTPAMMEGKVTNPSGIPFAEMSQIVGKQVNVIVAKGQTLMPDMVKTYA--GK

**viviparous-eelpout-S6†** mkcviftgllfvllcvdhmssaGE--SVVATQLIPMNTALTPAMMAGKVTNPSGIPFAEMSQIVGKQVNVIVAKGQTLMPDMVKTYA--GK

**viviparous-eelpout-S7†** mksvivtgllfvllcvdhm-taGQ--SVVATQLIPMNTALTLVMMEGKVTNPIGIPFAERDQIVGKQVNVIVAKGQTIMPGMVKTYA--GK

**viviparous-eelpout-S8†** -----------------hmssaGE--SVVATQLIPMNTALTLVMMEGKVTNPIGIPFGERDQIVGKQVNVIVAKGQTIMPDMVKTYA--GK

**viviparous-eelpout-S9†** ----iltgllfvllcvdhmssaGE--SVVATQLIPMNTALTLVMMEGKVTNPSGIPFAEKSQIVGKQVNVIVAKGQTIMPDMVKTYA--GK

**viviparous-eelpout-S10†** mksviltgllfvllcvdhmssaGE--SVVATQLIPMNTALTPAMMAGKVTNPSGIPFVEASQIVGKQVNVIVPKGQTIMPDMVKTYA--GK

**notched-fin eelpout-S3** mksviltgllfvllcvdhmssaGQ--SVVATQLIPMNTALTLAMMEGKVTNPSGIPFVEASQIVGKQVNVIVAKGQTIMPIMVKTYA--GK

**notched-fin eelpout-S4** ----------------------GQ--SVVATQLIPMNTALTPAMMEGKVTNPSGIPFVEASQIVGKQVNVIVAKGQTLMPDMVKTYA--GK

*:..:.**:.:***** :: :* * ** ***:*:*** * * *** : ::. **: * . *: ***
