# Supplementary material for: Antifreeze protein dispersion in eelpouts and related fishes reveals migration and climate alteration within the last 20 Ma
Source: PLoS One. 2020 Dec 15;15(12):e0243273. doi: 10.1371/journal.pone.0243273 (PMC7737890; doi:10.1371/journal.pone.0243273)
Supplement: S3 Fig — Sequences are named as in Fig 2. Grey highlighting indicates sequences that were determined by Edman degradation. The tandemers of Antarctic eelpout are lettered sequentially (e.g. Antarctic eelpout-Q3b is the second AFP domain in Antarctic eelpout-Q3). Variable residues are highlighted or coloured according to the phylogenetic tree (Fig 3) with conserved residues typical of QAE or SP variants highlighted cyan and yellow, respectively. Mutations that arose somewhere within Antarctic species are highlighted pink and residues within SAS-B that were not conserved when the QAE or SP groups arose are highlighted black. Other differences between SAS sequences are highlighted purple. Differences that do not correlate with these aforementioned groupings are highlighted grey. Red highlighting indicates shared differences between the signal peptides of the sequences from radiated shanny and rock gunnel. The black boxes and red boxes show residues involved in binding to the pyramidal plane and prism plane respectively, as in Fig 2. The signal peptide is in lowercase font. Italics indicate linkers between tandemers, internal dashes indicate gaps, whereas leading or trailing dashes indicate that the sequence is incomplete at either terminus. Identity, high similarity and low similarity between all AFPs (incomplete sequences ignored) is indicated at the bottom with asterisks, colons and periods, respectively. Residues with an inward pointing sidechain are indicated by “i” at the top. Asterisks denote sequences obtained by PCR in this study (S3 Table) and daggers denote those assembled from the SRA database (S4 Table). Accession numbers are listed in S4 and S5 Tables. (PDF) [file pone.0243273.s003.pdf]

```
                                                1   5   10      20      30      40      50      60      70
                                                    iiii  i     i   i      i      i    i   i    ii  i
notched-fin eelpout-Q1   mksviltgllfvllcvdhmssaNQ-ESVVAAVLIPINTALTVGMMTTRVVSPTGIPAEDIPRLISMQVNQAVPMGTTLMPDMVKFYCLCAPKN
viviparous-eelpout-Q1†   mksviltgllfvllcvdhmssaNQ-ESVVAAVLIPINTALTVGMMTTRVVSPTGIPAEDIPRLVSMQVNQAVPMGTTLMPDMVKFYCLCAPKN
ocean pout-Q1*           --------------cvdhmssaNQ-ESVVAATLIPINTALTVGMMTTRVVTPVGIPAEDIPRLVSMQVNQAVPMGTTLMPDMVKFYCLCAPKN
ocean pout-Q2            mksviltgllfvllcvdhmssaNQ-ESVVATQLIPINTALTLVMMTTRVIYPTGIPAEDIPRLVSMQVNQAVPMGTTLMPDMVKFYCLCAPKN
Atlantic wolffish-Q1     mksailtgllfvllcvdhmssaHQ-ASVVATQLIPINTALTLVMMTTRVVFPTGIPAEDIPRLVSMQVNRAVPMGTTLMPDMVKFYCLCAPKTQRW
Atlantic wolffish-Q2     ---ailtgllfvllcvdhmssaHQ-ASVVATQLIPINTALTLVMMTTAVVTPTGIPAEDIPRLVSMQVNRAVPMGTTLMPDMVKFYCLCAPKY
Alaskan ronquil-Q1*       -----------------nmssaK-GDSVVATQLIPINTALTLVMMGTADVTPTGIPAEDIPRLVGMQVNRAVLMGTTLMPDMVKFYCLC
ocean pout-Q3            mksviltgllfvllcvdhmssaNQ-ESVVATQLIPINTALTLVMMTTRVIYPTGIPAEDIPRLVSMQVNQAVPMGTTLLPDMVKGYPL-T
notched-fin eelpout-Q2   mksviltglffvllcvdhmssaNQ-ESVVAAVLIPINTALTVGMMTTRVVSPTGIPAEDIPRLISMQVNQVVPMGTTLMPDMVKGYAP-A
viviparous eelpout-Q2†   mksviltglffvllcvnhmssaNQ-ESVVAAVLIPINTALTVGMMTTRVVSPTGIPAEDIPRLISMQVNQVVPMGTTLMPDMVKGYAP-A
ocean pout-Q4*           --------------cvdhmssaNQ-ASVVATQLIPINTALTLVMMTTRVVTPMGIPAEDIPRLVSMQVNQAVACGTTLMPGMVKTYTP-AK
viviparous eelpout-Q3†   mksviltgllfvllcvdhmssaNQ-ASVVATQLIPINTALTLVMMKAKVVTPMGIPAEDIPRIVSMQVNQAVACGTTLMPGMVKTYTP-VK
notched-fin eelpout-Q3   mksviltgllfvllcvdhmssaNQ-ASVVATQLIPINTALTLVMMKAKVATPMGIPAEDIPRIVSMQVNQAVACGTTLMPGMVKTYTP-VK
ocean pout-Q5*           --------------cvdhmssaNQ-ASVVANQLIPINTALTLVMMRSEVVTPVGIPAEDIPRLVSMQVNRAVPLGTTLMPDMVKGYPP-A
radiated shanny-Q1*      mnsviftglvfvllcvdnmssaASGQSVVANQLIPINTALTLVMMRAEVVSPLGIPAEDIPRLVSLQVNRAVPLGTTLTAEMVKGYSP-AK
rock gunnel-Q1*          mnsailtgflfvllcvdnmfsaGSGKSVVANQLIPINTALTRIMMKAELVAPMGIPAEDIPRLVSLQVNRAVPMGTTLMPDMVKTYQP-AK
viviparous eelpout-Q4†   mksviltgllfvllcvdhmssaNQ-ASVVANQLIPINTALTLVMMRSEVVTPMGIPAVDIPRLVSMQVNRAVPLGTTLMPDMVKGYTP-A
notched-fin eelpout-Q4   mksviltgllfvllcvdhmssaNQ-ASVVANQLIPINTALTLVMMRAEVVTPMGIPAVDIPRLVSMQVNRAVPLGTTLMPEMVKGYTP-A
viviparous-eelpout-Q5†   mksviltgllfvllcvdhmssaDQ-ASVVANQLIPINTALTLVMMRAEVVTPMGIPAEDIPRLVSLQVNRAVPLGTTIMPDMVKGYAP-N
Canadian eelpout-Q1      ----------------------NK-ASVVANQLIPINTALTLVMMRAEVVTPAGIPAEDIPRLVGLQVNRAVLIGTTLMPDMVKGYAP-Q
spotted wolffish-Q1      mksailtgllfvllcvdhmssaHQ-ASIVANQLIPINTALTLIMMRAQVVTPLGIPAEDIPRIIGMQVNRAVALGTTLMPDMVKGYPP-N
spotted wolffish-Q2      mksailtgllfvllcvdhmssaHQ-ASIVANQLIPINTALTPIMTKAQVVTPLGIPAEDIPRIIGMQVNRAVALGTTLMPDMVKGYPP-N
P. brachycephalum-1†     mksvvltgllfvllcvdhmssaNK-ASVVANQLIPINTALTLVMMKAEVVTPMGIPAEDIPRLIGMQVNRAVPLGTTLMPDMVKMYCLCI
Antarctic eelpout-Q1     mksvvltgllfvllcvdhmssaNK-ASVVANQLIPINTALTLIMMKAEVVTPMGIPAEDIPRIIGMQVNRAVPLGTTLMPDMVKNY---EK
Antarctic eelpout-Q2     mksvvltgllfvllcvdhmssaNK-ASVVANQLIPINTALTLIMMKAEVVTPMGIPAEDIPKLMGMQVNRAVPLGTTLMPDMVKNY---EK
Antarctic eelpout-Q3a    mksvvltgllfvllcvdhmssaNK-ASVVANQLIPINTALTLIMMKAEVVTPMGIPAEEIPNLVGMQVNRAVPLGTTLMPDMVKNY---ED
Antarctic eelpout-Q4     mksvvltgllfvllcvdhmssaNK-ASVVANQLIPINTALTLVMMKAEVVTPMGIPAEEIPKLVGMQVNRAVPLGTTLMPDMVKNY---EK
Antarctic eelpout-Q3b          (tandemer)  GTTSPGLKSVVANQLIPINTALTLVMMKAEEVSPKGIPSEEISKLVGMQVNRAVPLGTTLMPDMVKNY---EK
P. brachycephalum-Q2†    mksvvltgllfvllcvdhmssaNK--SVVANQLIPINTALTLVMMKAEEVSPKGIPAEEIPRLVGMQVNRAVPLGTTLMPDMVKNY---QK
Antarctic eelpout-Q5b          (tandemer)  VTTCPGFKSAVANQLIPINTALTLMMMKAEEVSPKGIPAEEIPKLVGMQVNRAVYLDQTLMPDMVKNY---ED
Antarctic eelpout-Q6     -------------------------SVVANQLIPINTALTLVMMKAKEVSPKGIPAEEIPKLVGMQVNRAVYLDETLMPDMVKNY---EK
P. brachycephalum-Q3     ----------------------TK--SVVANQLIPINTALTLVMMKAEEVSPKGIPAEEIPRLVGMQVNRAVYLDETLMPDMVKNY---E
P. brachycephalum-Q4     mksvvltgllfvllcvdhmssaTK--SVVASQLIPINTALTPAMMKAKEVSPKGIPAEEMSKIVGMQVNRAVNLDETLMPDMVKTY---QK
ocean pout-Q6†           --------------cvdhmssaNQ-ASVVATQLIPINTALTLVMMKAKVVTPMGIPAEEIPQIVGLQVDRAVPLGTTLMPDMVKTYCA-AK
ocean pout-Q7            mksviltglflvllcvdhmssaNQ-ESVVATQLIPINTALTPIMMKGKVVTPMGIPFKEMSQIVGKQVNRAVPLGTTIMPEMVKGYAP-N
Antarctic eelpout-sasB   -------------------------GKSVVAKVKIPKGTVLTQDMLAVKAAEPMGIAAEDLCKMVGKTVTEDVEEDDSVMPEMVKGYCK-NKKC
wolf eel-sasB            -------------------------GKSVVAKVKIPKGTVLTQDMLAVKAAEPMGIAAEDLCKMVGKTVTEDVEEDDSIMPEMVKGYCK-NKKC
Antarctic eelpout-sasA   -------------------------GKSLVATVKIPKGTVLTQDMLTVKVAEPMGVAAEDIFQMVGKTVTKDVEEDGSLLPEVVDGYCK-KRKC
wolf eel-sasA            -------------------------GKSLVAKVKIPKGTVLTQDMLTVKVAEPMGIAAEDIFQMVGKTVTKDVEEDDSLLPEVVDGYCK-KRKC
Atlantic wolffish-S1     mksailtgllfvllcvdhmssaSQ--SVVATQLIPINTALTPIMMKGKVVNPAGIPFAEMSQIVGKQVNRPVAKDETLMPNMVKTYRA-AK
spotted wolffish-S1      mksailtgllfvllcvdhmssaSQ--SVVATQLIPINTALTPIMMKGKVVNPAGIPFAEMSQIVGKQVNRPVAKDETLMPNMVKTYRA-AK
spotted wolffish-S2      mksailtgllfvllcvdhmssaSQ--SVVATQLIPINTALTPAMMKGKVVSPAGIPFAEMSQIVGKQVNRPVAKDETLMPNMVKTYRA-AK
rock gunnel-S1*          mnsailtgflfvllcvdnmtsaGS--SVVASQLIPMNTALTPAMMKGVVVSPAGIPFAEMSRIVGKQVNQIVAKDQTLMPSMVKTYQP-AK
radiated shanny-S1*      mnsailtgflfvllcv-nmssaAQ--SVVATQLIPINTALTPAMMKGMDVNPSGIPFTEKSTLVGKQVNQPVVKGQTLMRNMVKP
ocean pout-S1            mksviltgllfvllcvdhm-taSQ--SVVATQLIPINTALTPAMMEGKVTNPIGIPFAEMSQIVGKQVNTPVAKGQTLMPNMVKTYVA-GK
ocean pout-S2*           --------------cvdhi-taSQ--SVVATRLIPMNTALTPAMMEGKVTNPIGIPFAEMSQIVGKQVNRIVAKGQTLMPNMVKTYAA-GK
ocean pout-S3            mksviltgllfvllcvdhm-taSQ--SVVATQLIPMNSALTPVMMEGKVTNPIGIPFAEMSQMVGKQVNRPVAKGQTIMPNMVKTYAA-GK
ocean pout-S4*           --------------cvdhm-taSQ--SVVATQLIPMNTALTPVMMEGKVTNPIGIPFAEMSQIVGKQVNTPVAKGQTIMPNMVKTYAA-GK
viviparous eelpout-S1    mkcviltgllfvllcvdhmssaGE--SVVATQLIPINTALTPAMMAGKVTNPSGIPFAEMSQIVGKQVNTPVAKGQTLMPDMVKTYVP-AK
notched-fin eelpout-S1   mksviltgllfvllcvdhmssaGE--SVVATQLIPINTALTPAMMEGKVTNPSGIPFAEMSQIVGKQVNTPVAKGQTLMPGMVKTYVP-AK
viviparous eelpout-S2    ----------------------GE--SVVATQLIPMNTALTLAMIEGKVTNPSGIPFAEKSQIVGKQVNTPVAKGQTLMPDMVKTYVP-AK
viviparous eelpout-S3†   mkcviltgllfvllcvdhmssaGE--SVVATQLIPMNTALTPAMMAGKVTNPSGIPFAEMLQIVGKQVNVIVAKGQTIMPNMVKTYAA-GK
viviparous eelpout-S4†   mkcviltgllfvllcvdhmssaGE--SVVATQLIPMNTALTLAMIEGKVTNPSGIPFAEKSQIVGKQVNVIVAKGQTIMPIMVKTYVP-AK
viviparous eelpout-S5†   mksviltgllfvllcvdhmssaGQ--SVVATQLIPMNTALTPAMMEGKVTNPSGIPFAEMSQIVGKQVNVIVPKGHTIMPIMVKTYA--GK
notched-fin eelpout-S2   mksviltgllfvllcvdhmssaGE--SVVATQLIPINTALTPAMMEGKVTNPSGIPFAEMSQIVGKQVNVIVAKGQTLMPDMVKTYA--GK
viviparous-eelpout-S6†   mkcviftgllfvllcvdhmssaGE--SVVATQLIPMNTALTPAMMAGKVTNPSGIPFAEMSQIVGKQVNVIVAKGQTLMPDMVKTYA--GK
viviparous-eelpout-S7†   mksvivtgllfvllcvdhm-taGQ--SVVATQLIPMNTALTLVMMEGKVTNPIGIPFAERDQIVGKQVNVIVAKGQTIMPGMVKTYA--GK
viviparous-eelpout-S8†   -----------------hmssaGE--SVVATQLIPMNTALTLVMMEGKVTNPIGIPFGERDQIVGKQVNVIVAKGQTIMPDMVKTYA--GK
viviparous-eelpout-S9†   ----iltgllfvllcvdhmssaGE--SVVATQLIPMNTALTLVMMEGKVTNPSGIPFAEKSQIVGKQVNVIVAKGQTIMPDMVKTYA--GK
viviparous-eelpout-S10†  mksviltgllfvllcvdhmssaGE--SVVATQLIPMNTALTPAMMAGKVTNPSGIPFVEASQIVGKQVNVIVPKGQTIMPDMVKTYA--GK
notched-fin eelpout-S3   mksviltgllfvllcvdhmssaGQ--SVVATQLIPMNTALTLAMMEGKVTNPSGIPFVEASQIVGKQVNVIVAKGQTIMPIMVKTYA--GK
notched-fin eelpout-S4   ----------------------GQ--SVVATQLIPMNTALTPAMMEGKVTNPSGIPFVEASQIVGKQVNVIVAKGQTLMPDMVKTYA--GK
                         *:..:.**:.:***** :: :*    * **  ***:*:**:*   *          * ***  :    :.:  **:  *  .  *:    ***
```
