# Supplementary material for: Antifreeze protein dispersion in eelpouts and related fishes reveals migration and climate alteration within the last 20 Ma
Source: PLoS One. 2020 Dec 15;15(12):e0243273. doi: 10.1371/journal.pone.0243273 (PMC7737890; doi:10.1371/journal.pone.0243273)
Supplement: S4 Fig — Sequences are named and variable nucleotides are highlighted as in Fig 2. Red highlighting indicates shared differences between the first exons of radiated shanny and rock gunnel and these exons were excluded prior to generating all but the exon 1 phylogenetic tree as they may have been homogenized by exon shuffling. The translation of notched-fin eelpout-Q1 has black boxes and red boxes showing residues involved in binding to the pyramidal plane and prism plane respectively, as in Fig 2. The signal peptide is in lowercase font. Internal dashes indicate gaps, whereas leading or trailing dashes indicate that the sequence is incomplete at the respective terminus. Intronic sequences are not shown for genomic clones but an arrow indicates where an intron is found. The 3′ splice junction of ocean pout-Q2 was originally predicted based on SP isoforms but has been adjusted by three bases (lower-case font) to match QAE-type cDNAs. The 3ʹ end of P. brachycephalum-Q1 (italics) and the linker sequences between Antarctic eelpout tandemers (not shown) were excluded from the phylogenetic analysis as they are not homologous to the other AFP sequences. These nucleotide sequences are unambiguously accessed through the protein accession numbers in S4 and S5 Tables as some of the nucleotide sequences encode multiple AFPs. (DOCX) [file pone.0243273.s004.docx]

**notched-fin eelpout-Q1** m k s v i l t g l l f v l l c v d h m s

**notched-fin eelpout-Q1** ATGAAGTCAGTCATTTTAACTGGTTTGCTTTTCGTCCTCCTTTGTGTCGACCACATGAGT

**viviparous eelpout-Q1** ATGAAGTCAGTCATTTTAACTGGTTTGCTCTTCGTCCTCCTTTGTGTCGACCACATGAGT

**ocean pout-Q1**  -----------------------------------------TTGTGTCGACCACATGAGT

**ocean pout-Q2** ATGAAGTCAGTCATTTTAACTGGTTTGCTTTTCGTCCTCCTTTGTGTCGACCACATGAGT

**Atlantic wolffish-Q1** ATGAAGTCAGCTATTTTAACTGGTTTGCTTTTCGTCCTCCTTTGTGTCGACCACATGAGT

**Atlantic wolffish-Q2** --------AGCTATTTTAACTGGTTTGCTTTTCGTCCTCCTTTGTGTCGACCACATGAGT

**Alaskan ronquil-1**  ---------------------------------------------------AACATGAGT

**ocean pout-Q3**  ATGAAGTCAGTCATTTTAACTGGTTTGCTTTTCGTCCTCCTTTGTGTCGACCACATGAGT

**notched-fin eelpout-Q2** ATGAAGTCAGTCATTTTAACTGGTTTGTTCTTCGTCCTCCTTTGTGTCGACCACATGAGT

**viviparous eelpout-Q2** ATGAAGTCAGTCATTTTAACTGGTTTGTTCTTCGTCCTCCTTTGTGTCAACCACATGAGT

**ocean pout-Q4**  -----------------------------------------TTGTGTCGACCACATGAGT

**viviparous eelpout-Q3** ATGAAGTCAGTCATTTTAACTGGTTTGCTTTTCGTCCTCCTTTGTGTCGACCACATGAGT

**notched-fin eelpout-Q3** ATGAAGTCAGTCATTTTAACTGGTTTGCTTTTCGTCCTCCTTTGTGTCGACCACATGAGT

**ocean pout-Q5**  -----------------------------------------TTGTGTCGACCACATGAGT

**radiated shanny-Q1** ATGAATTCAGTTATTTTTACTGGTTTGGTCTTCGTCCTCCTTTGTGTCGACAACATGAGT

**rock gunnel-Q1**  ATGAATTCAGCTATTTTAACTGGTTTCCTTTTCGTCCTCCTTTGTGTCGACAACATGACT

**viviparous eelpout-Q4**  ATGAAGTCAGTCATTTTAACTGGTTTGCTTTTCGTCCTCCTTTGTGTCGACCACATGAGT

**notched-fin eelpout-Q4** ATGAAGTCAGTCATTTTAACTGGTTTGCTTTTCGTCCTCCTTTGTGTCGACCACATGAGT

**viviparous eelpout-Q5** ATGAAGTCAGTCATTTTAACTGGTTTGCTTTTCGTCCTCCTTTGTGTCGACCACATGAGT

**spotted wolffish-Q1** ATGAAGTCAGCTATTTTAACTGGTTTGCTTTTCGTCCTCCTTTGTGTCGACCACATGAGT

**spotted wolffish-Q2** ATGAAGTCAGCTATTTTAACTGGTTTGCTTTTCGTCCTCCTTTGTGTCGACCACATGAGT

***P. brachycephalum-*Q1** ATGAAGTCAGTTGTTTTAACTGGTTTGCTGTTCGTCCTCCTTTGTGTCGACCACATGAGT

**Antarctic eelpout-Q1** ATGAAGTCAGTTGTTTTAACTGGTTTGCTGTTCGTCCTCCTTTGTGTCGACCACATGAGT

**Antarctic eelpout-Q2** ATGAAGTCAGTTGTTTTAACTGGTTTGCTGTTCGTCCTCCTTTGTGTCGACCACATGAGT

**Antarctic eelpout-Q3a** ATGAAGTCAGTTGTTTTAACTGGTTTGCTGTTCGTCCTCCTTTGTGTCGACCACATGAGT

**Antarctic eelpout-Q4** ATGAAGTCAGTTGTTTTAACTGGTTTGCTGTTCGTCCTCCTTTGTGTCGACCACATGAGT

**Antarctic eelpout-Q3b** ------------------------------------------------------------

***P. brachycephalum*-Q2**  ATGAAGTCAGTTGTTTTAACTGGTTTGCTGTTCGTCCTCCTTTGTGTCGACCACATGAGT

**Antarctic eelpout-Q5b** ------------------------------------------------------------

**Antarctic eelpout-Q6** ------------------------------------------------------------

***P. brachycephalum*-Q4** ATGAAGTCAGTTGTTTTAACTGGTTTGCTGTTCGTCCTCCTTTGTGTCGACCACATGAGT

**ocean pout-Q6** -----------------------------------------TTGTGTCGACCACATGAGT

**ocean pout-Q7** ATGAAGTCAGTCATTTTAACTGGTTTGTTTCTTGTCCTCCTTTGTGTCGACCACATGAGT

**Antarctic eelpout-sasB** AATAATTCCGTTATTTTCACCAGTTTGTTTTCCGTTTTCTTTTCTGCAGACAAAATGCCG

**wolf eel-sasB** AATAATTCTGCTATTTTCACCGGTT-------CGTTTTCTTTTCTTTGGACAAAATGCCT

**Antarctic eelpout-sasA** -----------------------------------TTTGTTTTCTGTGGACAAAATGCCG

**wolf eel-sasA** AATAATTCCGTTAATTGAACCGGTTTGTTTTCCGTTTTCTTTTCTGTGGGCAAAATGCCG

**Atlantic wolffish-S1** ATGAAGTCAGCTATTTTAACCGGTTTGCTTTTCGTCCTCCTTTGTGTCGACCACATGAGT

**spotted wolffish-S1** ATGAAGTCAGCTATTTTAACCGGTTTGCTTTTCGTCCTCCTTTGTGTCGACCACATGAGT

**spotted wolffish-S2** ATGAAGTCAGCTATTTTAACCGGTTTGCTTTTCGTCCTCCTTTGTGTCGACCACATGAGT

**rock gunnel-S1** ATGAATTCAGCTATTTTAACTGGTTTCCTTTTCGTCCTCCTTTGTGTCGACAACATGACT

**radiated shanny-S1** ATGAATTCAGCTATTTTAACTGGTTTTCTTTTCGTCCTCCTTTGTGTC---AACATGAGT

**ocean pout-S1** ATGAAGTCCGTTATTTTAACCGGTTTGCTCTTCGTCCTCCTTTGTGTCGACCACATGA--

**ocean pout-S2** -----------------------------------------TTGTGTCGACCACATCA--

**ocean pout-S3** ATGAAGTCCGTTATTTTAACCGGTTTGCTTTTCGTCCTCCTTTGTGTCGACCACATGA—-

**ocean pout-S4** -----------------------------------------TTGTGTCGACCACATGA--

**viviparous eelpout-S1** ATGAAGTGCGTTATTTTAACCGGTTTGCTTTTCGTCCTCCTTTGTGTCGACCACATGAGT

**notched-fin eelpout-S1** ATGAAGTCCGTTATTTTAACCGGTTTGCTTTTCGTCCTCCTTTGTGTCGACCACATGAGT

**viviparous eelpout-S2** ------------------------------------------------------------

**viviparous eelpout-S3** ATGAAGTGCGTTATTTTAACCGGTTTGCTTTTCGTCCTCCTTTGTGTCGACCACATGAGT

**viviparous eelpout-S4** ATGAAGTGCGTTATTTTAACCGGTTTGCTTTTCGTCCTCCTTTGTGTCGACCACATGAGT

**viviparous eelpout-S5** ATGAAGTCCGTTATTTTAACCGGTTTGCTTTTCGTCCTCCTTTGTGTCGACCACATGAGT

**notched-fin eelpout-S2** ATGAAGTCCGTTATTTTAACCGGTTTGCTTTTCGTCCTCCTTTGTGTCGACCACATGAGT

**viviparous eelpout-S6** ATGAAGTGCGTTATTTTTACCGGTTTGCTTTTCGTCCTCCTTTGTGTCGACCACATGAGT

**viviparous eelpout-S7** ATGAAGTCCGTTATTGTAACCGGTTTGCTTTTCGTCCTCCTTTGTGTCGACCACATGA--

**viviparous eelpout-S8** -------------------------------------------------ACCACATGAGT

**viviparous eelpout-S9** ------------ATTTTAACCGGTTTGCTTTTCGTCCTCCTTTGTGTCGACCACATGAGT

**viviparous eelpout-S10** ATGAAGTCCGTTATTTTAACCGGTTTGCTTTTCGTCCTCCTTTGTGTCGACCACATGAGT

**notched-fin eelpout-S3** ATGAAGTCCGTTATTTTAACCGGTTTGCTTTTCGTCCTCCTTTGTGTCGACCACATGAGT

**notched-fin eelpout-S4** ------------------------------------------------------------

**notched-fin eelpout-Q1** s a N Q - E S V V A A V L I P I N T A L

**notched-fin eelpout-Q1** TCAGCCAACCAG---GAGTCCGTGGTGGCCGCCGTTCTGATCCCCATAAATACAGCCCTG

**viviparous eelpout-Q1**  TCAGCCAACCAG---GAGTCCGTGGTGGCCGCCGTTCTGATCCCCATAAATACAGCCCTG

**ocean pout-Q1** TCAGCCAACCAG---GAGTCCGTGGTGGCCGCCACGCTGATCCCCATAAATACTGCCCTG

**ocean pout-2** TCAGCCAACCAG---gagTCCGTGGTGGCCACCCAGCTGATCCCCATAAATACTGCCCTG

**Atlantic wolffish-Q1** TCAGCCCACCAG---GCGTCCGTGGTGGCCACCCAGCTGATCCCCATAAATACTGCCCTG

**Atlantic wolffish-Q2** TCAGCCAACCAG---GCGTCCGTGGTGGCCACCCAGCTGATCCCCATAAATACTGCCCTG

**Alaskan ronquil-Q1**  TCAGCCAAG---GGTGATTCCGTGGTGGCCACCCAGCTGATCCCCATAAATACTGCCCTG

**ocean pout-Q3**  TCAGCCAACCAG---GAGTCCGTGGTGGCCACCCAGCTGATCCCCATAAATACTGCCCTG

**notched-fin eelpout-Q2** TCAGCCAACCAG---GAGTCCGTGGTGGCCGCCGTTCTGATCCCCATAAATACTGCCCTG

**viviparous eelpout-Q2** TCAGCCAACCAG---GAGTCCGTGGTGGCCGCCGTTCTGATCCCCATAAATACTGCCCTG

**ocean pout-Q4**  TCAGCCAACCAG---GCGTCCGTGGTGGCCACCCAGCTGATCCCCATAAATACTGCCCTG

**viviparous eelpout-Q3** TCAGCCAACCAG---GCGTCCGTGGTGGCCACCCAGCTGATCCCCATAAATACTGCCCTG

**notched-fin eelpout-Q3** TCAGCCAACCAG---GCGTCCGTGGTGGCCACCCAGCTGATCCCCATAAATACTGCCCTG

**ocean pout-Q5**  TCAGCCAACCAG---GCGTCCGTGGTGGCCAACCAGCTGATCCCCATAAATACTGCCCTG

**radiated shanny-Q1** TCAGCCGCCTCGGGTCAGTCCGTGGTGGCCAACCAGCTGATCCCCATCAATACTGCCCTG

**rock gunnel-Q1** TCAGCCGGCTCGGGTAAATCCGTGGTGGCCAACCAGCTGATCCCCATAAATACTGCCCTG

**viviparous eelpout-Q4** TCAGCCAACCAG---GCGTCCGTGGTGGCCAACCAGCTGATCCCCATAAATACTGCCCTG

**notched-fin eelpout-Q4** TCAGCCAACCAG---GCGTCCGTGGTGGCCAACCAGCTGATCCCCATAAATACTGCCCTG

**viviparous eelpout-Q5** TCAGCCGACCAG---GCGTCCGTGGTGGCCAACCAGCTGATCCCCATAAATACTGCCCTG

**spotted wolffish-Q1** TCAGCCCACCAG---GCGTCCATTGTGGCCAACCAGCTGATCCCCATAAATACTGCCCTG

**spotted wolffish-Q2** TCAGCCCACCAG---GCGTCCATTGTGGCCAACCAGCTGATCCCCATAAATACTGCCCTG

***P. brachycephalum-*Q1** TCAGCCAACAAG---GCGTCCGTGGTGGCCAACCAGCTGATCCCCATAAATACTGCCCTG

**Antarctic eelpout-Q1** TCAGCCAACAAG---GCGTCCGTGGTGGCCAACCAGCTGATCCCCATAAATACTGCCCTG

**Antarctic eelpout-Q2** TCAGCCAACAAG---GCGTCCGTGGTGGCCAACCAGCTGATCCCCATAAATACTGCCCTG

**Antarctic eelpout-Q3a** TCAGCCAACAAG---GCGTCCGTGGTGGCCAACCAGCTGATCCCCATAAATACTGCCCTG

**Antarctic eelpout-Q4** TCAGCCAACAAG---GCGTCCGTGGTGGCCAACCAGCTGATCCCCATAAATACTGCCCTG

**Antarctic eelpout-Q3b** ----tandemer------TCCGTGGTGGCCAACCAGCTGATCCCCATAAATACTGCCCTG

***P. brachycephalum*-Q2** TCAGCCAACAAG------TCCGTGGTGGCCAACCAGCTGATCCCCATAAATACTGCCCTG

**Antarctic eelpout-Q5b** ----tandemer------TCCGCGGTGGCCAACCAGCTGATCCCCATAAATACTGCCCTG

**Antarctic eelpout-Q6** likely tandemer---TCCGTGGTGGCCAACCAGCTGATCCCCATAAATACTGCCCTG

***P. brachycephalum*-Q4** TCAGCCACCAAG------TCCGTGGTGGCCAGCCAGCTGATCCCCATAAATACTGCCCTG

**ocean pout-Q6** TCAGCCAACCAG---GCGTCCGTGGTGGCCACCCAGCTGATCCCCATAAATACTGCCCTG

**ocean pout-Q7** TCAGCCAACCAG---GAGTCCGTGGTGGCCACCCAGCTGATCCCCATAAATACTGCCCTG

**Antarctic eelpout-sasB** TTAACGTTCGAGGGTAAATCTGTGGTGGCCAAGGTCAAGATCCCCAAAGGAACCGTCCTG

**wolf eel-sasB** TTAACGTTCGAGGGTAAATCTGTGGTGGCCAAGGTCAAGATCCCCAAAGGAACTGTCCTG

**Antarctic eelpout-sasA** TTAACGTTCGAGGGTAAGTCCTTGGTGGCCACGGTCAAGATCCCCAAAGGCACCGTCCTG

**wolf eel-sasA** TTAACGTTCGAGGGTAAGTCCTTGGTGGCCAAGGTCAAGATCCCCAAAGGAACCGTCCTG

**Atlantic wolffish-S1** TCAGCCAGCCAG------TCCGTGGTGGCCACCCAGCTGATCCCCATAAATACTGCCCTG

**spotted wolffish-S1** TCAGCCAGCCAG------TCCGTGGTGGCCACCCAGCTGATCCCCATAAATACTGCCCTG

**spotted wolffish-S2** TCAGCCAGCCAG------TCCGTGGTGGCCACCCAGCTGATCCCCATAAATACTGCCCTG

**rock gunnel-S1** TCAGCCGGCTCG------TCCGTGGTGGCCAGCCAGCTGATCCCCATGAATACTGCCCTG

**radiated shanny-S1** TCAGCCGCCCAG------TCCGTGGTGGCCACTCAGCTGATCCCCATAAATACTGCCCTG

**ocean pout-S1** -CAGCCAGCCAG------TCCGTGGTGGCCACCCAGCTGATCCCCATAAATACTGCCCTG

**ocean pout-S2** -CAGCCAGCCAG------TCCGTGGTGGCCACCCGGCTGATCCCCATGAATACTGCCCTG

**ocean pout-S3** -CAGCCAGCCAG------TCCGTGGTGGCCACCCAGCTGATCCCCATGAATAGTGCCCTG

**ocean pout-S4** -CAGCCAGCCAG------TCCGTGGTGGCCACCCAGCTGATCCCCATGAATACTGCCCTG

**viviparous eelpout-S1** TCAGCCGGCGAG------TCCGTGGTGGCCACCCAGCTGATCCCCATAAATACTGCCCTG

**notched-fin eelpout-S1** TCAGCCGGCGAG------TCCGTGGTGGCCACCCAGCTGATCCCCATAAATACTGCCCTG

**viviparous eelpout-S2** ------GGCGAG------TCCGTGGTGGCCACCCAGCTGATCCCCATGAATACTGCCCTG

**viviparous eelpout-S3** TCAGCCGGCGAG------TCCGTGGTGGCCACCCAGCTGATCCCCATGAATACTGCCCTG

**viviparous eelpout-S4** TCAGCCGGCGAG------TCCGTGGTGGCCACCCAGCTGATCCCCATGAATACTGCCCTG

**viviparous eelpout-S5** TCAGCCGGCCAG------TCCGTGGTGGCCACCCAGCTGATCCCCATGAATACTGCCCTG

**notched-fin eelpout-S2** TCAGCCGGCGAG------TCCGTGGTGGCCACCCAGCTGATCCCCATAAATACTGCCCTG

**viviparous eelpout-S6** TCAGCCGGCGAG------TCCGTGGTGGCCACCCAGCTGATCCCCATGAATACTGCCCTG

**viviparous eelpout-S7** -CAGCCGGCCAG------TCCGTGGTGGCCACCCAGCTGATCCCCATGAATACTGCCCTG

**viviparous eelpout-S8** TCAGCCGGCGAG------TCCGTGGTGGCCACCCAGCTGATCCCCATGAATACTGCCCTG

**viviparous eelpout-S9** TCAGCCGGCGAG------TCCGTGGTGGCCACCCAGCTGATCCCCATGAATACTGCCCTG

**viviparous eelpout-S10** TCAGCCGGCGAG------TCCGTGGTGGCCACCCAGCTGATCCCCATGAATACTGCCCTG

**notched-fin eelpout-S3** TCAGCCGGCCAG------TCCGTGGTGGCCACCCAGCTGATCCCCATGAATACTGCCCTG

**notched-fin eelpout-S4** ------GGCCAG------TCCGTGGTGGCCACCCAGCTGATCCCCATGAATACTGCCCTG

**notched-fin eelpout-Q1** T V G M M T T R V V S P T G I P A E D I

**notched-fin eelpout-Q1** ACAGTGGGGATGATGACGACACGGGTGGTCTCCCCAACGGGCATCCCCGCCGAGGACATT

**viv-eelpout-1** ACAGTGGGGATGATGACGACACGGGTGGTCTCCCCAACGGGCATCCCCGCCGAGGACATT

**ocean pout-Q1** ACTGTGGGGATGATGACGACACGGGTGGTCACCCCAGTGGGCATCCCCGCCGAGGACATT

**ocean pout-2** ACTCTGGTGATGATGACGACACGGGTTATCTACCCAACGGGCATCCCCGCCGAGGACATT

**Atlantic wolffish-Q1** ACTCTGGTGATGATGACGACCAGGGTGGTCTTCCCAACGGGCATCCCCGCCGAGGACATT

**Atlantic wolffish-Q2** ACTCTGGTGATGATGACGACAGCGGTGGTCACCCCAACGGGCATCCCCGCCGAGGACATT

**Alaskan ronquil-Q1**  ACTCTGGTGATGATGGGGACAGCGGACGTCACCCCAACGGGCATCCCCGCCGAGGACATT

**ocean pout-Q3**  ACTCTGGTGATGATGACGACACGGGTTATCTACCCAACGGGCATCCCCGCCGAGGACATT

**notched-fin eelpout-Q2** ACTGTGGGGATGATGACGACACGGGTGGTCTCCCCAACGGGCATCCCCGCCGAGGACATT

**viviparous eelpout-Q2** ACTGTGGGGATGATGACGACACGGGTGGTCTCCCCAACGGGCATCCCCGCCGAGGACATT

**ocean pout-Q4**  ACTCTGGTGATGATGAGGTCGAAGGTGGTCACCCCAATGGGCATCCCCGCCGAGGACATT

**viviparous eelpout-Q3** ACTCTGGTGATGATGAAGGCGAAGGTGGTCACCCCAATGGGCATCCCCGCCGAGGACATT

**notched-fin eelpout-Q3** ACTCTGGTGATGATGAAGGCGAAGGTGGCCACCCCAATGGGCATCCCCGCCGAGGACATT

**ocean pout-Q5**  ACTCTGGTGATGATGAGGTCGGAGGTGGTCACCCCAGTGGGCATCCCCGCCGAAGACATT

**radiated shanny-Q1** ACTCTGGTGATGATGAGGGCGGAGGTGGTCTCCCCATTGGGCATCCCCGCCGAGGACATT

**rock gunnel-Q1** ACTCGGATCATGATGAAGGCGGAGTTGGTCGCCCCAATGGGCATCCCCGCCGAGGACATT

**viviparous eelpout-Q4** ACTCTGGTGATGATGAGGTCGGAGGTGGTCACCCCAATGGGCATCCCCGCCGTGGACATT

**notched-fin eelpout-Q4** ACTCTGGTGATGATGAGGGCGGAGGTGGTCACCCCAATGGGCATCCCCGCCGTGGACATT

**viviparous eelpout-Q5** ACTCTGGTGATGATGAGGGCGGAGGTGGTCACCCCAATGGGCATCCCCGCCGAGGACATT

**spotted wolffish-Q1** ACTCTGATAATGATGAGGGCGCAGGTGGTCACCCCATTGGGCATCCCTGCCGAGGACATT

**spotted wolffish-Q2** ACTCCGATAATGACGAAGGCGCAGGTGGTCACCCCATTGGGCATCCCTGCCGAGGACATT

***P. brachycephalum*-Q1** ACTCTAGTGATGATGAAGGCGGAGGTGGTCACCCCAATGGGCATCCCCGCCGAGGACATT

**Antarctic eelpout-Q1** ACTCTGATAATGATGAAGGCGGAGGTGGTCACCCCAATGGGCATCCCCGCCGAGGACATT

**Antarctic eelpout-Q2** ACTCTGATAATGATGAAGGCGGAGGTGGTCACCCCAATGGGCATCCCTGCCGAGGACATC

**Antarctic eelpout-Q3a** ACTCTGATAATGATGAAGGCGGAGGTGGTCACCCCAATGGGCATCCCCGCCGAGGAGATC

**Antarctic eelpout-Q4** ACTCTAGTGATGATGAAGGCGGAGGTGGTCACCCCAATGGGCATCCCTGCCGAGGAGATC

**Antarctic eelpout-Q3b** ACTCTAGTGATGATGAAGGCGGAGGAAGTCAGCCCAAAGGGCATCCCTTCCGAGGAGATC

***P. brachycephalum*-Q2** ACTCTAGTGATGATGAAGGCGGAGGAAGTCAGCCCAAAGGGCATCCCTGCCGAGGAGATC

**Antarctic eelpout-Q5b** ACTCTAATGATGATGAAGGCGGAGGAAGTCAGCCCAAAGGGCATCCCTGCCGAGGAGATC

**Antarctic eelpout-Q6** ACTCTAGTGATGATGAAGGCGAAGGAAGTCAGCCCAAAGGGCATCCCTGCCGAGGAGATC

***P. brachycephalum*-Q4** ACTCCAGCGATGATGAAGGCGAAGGAAGTCAGCCCAAAGGGCATCCCTGCCGAGGAGATG

**ocean pout-Q6** ACTCTGGTGATGATGAAGGCGAAGGTGGTCACCCCAATGGGCATCCCCGCCGAGGAAATA

**ocean pout-Q7** ACTCCGATAATGATGAAGGGGAAGGTGGTCACCCCAATGGGCATCCCGTTCAAGGAGATG

**Antarctic eelpout-sasB** ACTCAGGACATGTTGGCGGTGAAGGCGGCCGAGCCGATGGGTATCGCGGCCGAGGACTTG

**wolf eel-sasB** ACTCAGGACATGTTGGCGGTGAAGGCGGCCGAGCCGATGGGTATCGCGGCCGAGGACTTG

**Antarctic eelpout-sasA** ACTCAGGACATGTTGACGGTGAAGGTGGCCGAGCCGATGGGCGTCGCGGCCGAGGACATC

**wolf eel-sasA** ACTCAGGACATGTTGACGGTGAAGGTGGCTGAGCCGATGGGCATCGCGGCCGAGGACATC

**Atlantic wolffish-S1** ACTCCGATAATGATGAAGGGGAAGGTGGTCAACCCAGCGGGCATCCCGTTCGCGGAGATG

**spotted wolffish-S1** ACTCCGATAATGATGAAGGGGAAGGTGGTCAACCCAGCGGGCATCCCGTTCGCGGAGATG

**spotted wolffish-S2** ACTCCGGCGATGATGAAGGGGAAGGTGGTCAGCCCAGCGGGCATCCCGTTCGCGGAGATG

**rock gunnel-S1** ACTCCGGCGATGATGAAGGGGGTTGTGGTCAGCCCAGCAGGCATCCCGTTCGCAGAGATG

**radiated shanny-S1** ACTCCGGCAATGATGAAGGGGATGGATGTCAACCCAAGCGGCATCCCGTTCACGGAGAAG

**ocean pout-S1** ACTCCGGCGATGATGGAGGGGAAGGTGACCAACCCAATAGGCATCCCGTTCGCGGAGATG

**ocean pout-S2** ACTCCGGCGATGATGGAGGGGAAGGTGACCAACCCAATAGGCATCCCGTTCGCGGAGATG

**ocean pout-S3** ACTCCGGTGATGATGGAGGGGAAGGTGACCAACCCAATAGGCATCCCGTTCGCGGAGATG

**ocean pout-S4** ACTCCGGTGATGATGGAGGGGAAGGTGACCAACCCAATAGGCATCCCGTTCGCGGAGATG

**viviparous eelpout-S1** ACTCCGGCGATGATGGCGGGGAAGGTGACCAACCCAAGCGGCATCCCGTTCGCGGAGATG

**notched-fin eelpout-S1** ACTCCGGCGATGATGGAGGGGAAGGTGACCAACCCAAGCGGCATCCCGTTCGCGGAGATG

**viviparous eelpout-S2** ACTCTGGCGATGATAGAGGGGAAGGTGACCAACCCAAGCGGCATCCCGTTCGCAGAGAAG

**viviparous eelpout-S3** ACTCCGGCGATGATGGCGGGGAAGGTGACCAACCCAAGCGGCATCCCGTTCGCGGAGATG

**viviparous eelpout-S4** ACTCTGGCGATGATAGAGGGGAAGGTGACCAACCCAAGCGGCATCCCGTTCGCAGAGAAG

**viviparous eelpout-S5** ACTCCGGCGATGATGGAGGGGAAGGTGACCAACCCAAGCGGCATCCCGTTCGCGGAGATG

**notched-fin eelpout-S2** ACTCCGGCGATGATGGAGGGGAAGGTGACCAACCCAAGCGGCATCCCGTTCGCGGAGATG

**viviparous eelpout-S6** ACTCCGGCGATGATGGCGGGGAAGGTGACCAACCCAAGCGGCATCCCGTTCGCGGAGATG

**viviparous eelpout-S7** ACTCTGGTGATGATGGAGGGGAAGGTGACCAACCCAATAGGCATCCCGTTCGCGGAGAGG

**viviparous eelpout-S8** ACTCTGGTGATGATGGAGGGGAAGGTGACCAACCCAATAGGCATCCCGTTCGGGGAGAGG

**viviparous eelpout-S9** ACTCTGGTGATGATGGAGGGGAAGGTGACCAACCCAAGCGGCATCCCGTTCGCGGAGAAG

**viviparous eelpout-S10** ACTCTGGCGATGATGGCGGGGAAGGTGACCAACCCAAGCGGCATCCCGTTCGTGGAGGCG

**notched-fin eelpout-S3** ACTCTGGCGATGATGGAGGGGAAGGTGACCAACCCAAGTGGCATCCCGTTCGTGGAGGCG

**notched-fin eelpout-S4** ACTCCGGCGATGATGGAGGGGAAGGTGACCAACCCAAGCGGCATCCCGTTCGTGGAGGCG

**notched-fin eelpout-Q1** P R L I S M Q V N Q A V P M G T T L M P

**notched-fin eelpout-Q1** CCCCGATTAATCTCAATGCAAGTGAACCAGGCAGTGCCGATGGGCACAACCCTCATGCCA

**viviparous eelpout-Q1** CCCCGATTAGTCTCAATGCAAGTGAACCAGGCAGTGCCGATGGGCACAACCCTCATGCCA

**ocean pout-Q1** CCCCGATTAGTCTCAATGCAAGTGAACCAGGCAGTGCCGATGGGCACAACCCTCATGCCA

**ocean pout-2** CCCCGATTAGTCTCAATGCAAGTGAACCAGGCAGTGCCGATGGGCACAACCCTCATGCCA

**Atlantic wolffish-Q1** CCCCGATTAGTCTCAATGCAAGTGAACAGGGCAGTGCCGATGGGCACAACCCTCATGCCA

**Atlantic wolffish-Q2** CCCCGATTAGTCTCAATGCAAGTGAACAGGGCAGTGCCGATGGGCACAACCCTCATGCCA

**Alaskan ronquil-Q1**  CCCCGATTAGTCGGAATGCAAGTGAACAGGGCAGTGCTGATGGGCACAACCCTCATGCCA

**ocean pout-Q3**  CCCCGATTAGTCTCAATGCAAGTGAACCAGGCAGTGCCGATGGGCACAACCCTCCTGCCA

**notched-fin eelpout-Q2** CCCCGATTAATCTCAATGCAAGTGAACCAGGTAGTGCCGATGGGCACAACCCTCATGCCA

**viviparous eelpout-Q2** CCCCGATTAATCTCAATGCAAGTGAACCAGGTAGTGCCGATGGGCACAACCCTCATGCCA

**ocean pout-Q4**  CCCCGATTAGTCTCAATGCAAGTGAACCAGGCAGTGGCGTGTGGCACAACCCTCATGCCA

**viviparous eelpout-Q3** CCCCGAATAGTCTCAATGCAAGTGAACCAGGCAGTGGCGTGTGGCACAACCCTCATGCCA

**notched-fin eelpout-Q3** CCCCGAATAGTCTCAATGCAAGTGAACCAGGCAGTGGCGTGTGGCACAACCCTCATGCCA

**ocean pout-Q5**  CCCCGATTAGTCTCAATGCAAGTGAACAGGGCAGTGCCGTTGGGCACAACCCTCATGCCA

**radiated shanny-Q1** CCCCGACTAGTCAGTCTGCAAGTGAACAGGGCAGTGCCGCTGGGCACAACCCTCACGGCA

**rock gunnel-Q1** CCCCGACTAGTCAGTCTGCAAGTCAACAGGGCAGTGCCGATGGGCACAACCCTCATGCCA

**viviparous eelpout-Q4** CCCCGATTAGTCTCAATGCAAGTGAACAGGGCAGTGCCGTTGGGCACAACCCTCATGCCA

**notched-fin eelpout-Q4** CCCCGATTAGTCTCAATGCAAGTGAACAGGGCAGTGCCGTTGGGCACAACCCTCATGCCA

**viviparous eelpout-Q5** CCCCGATTAGTCTCATTGCAAGTGAACAGGGCAGTGCCGTTGGGCACAACCATCATGCCA

**spotted wolffish-Q1** CCCCGAATAATCGGAATGCAAGTGAACAGGGCAGTGGCGTTGGGCACAACCCTCATGCCA

**spotted wolffish-Q2** CCCCGAATAATCGGAATGCAAGTGAACAGGGCAGTGGCGTTGGGCACAACCCTCATGCCA

***P. brachycephalum*-Q1** CCCCGACTAATCGGAATGCAAGTGAACAGGGCAGTGCCGTTGGGCACAACCCTCATGCCA

**Antarctic eelpout-Q1** CCCCGAATAATCGGAATGCAAGTGAACAGGGCAGTGCCGTTGGGCACAACCCTCATGCCA

**Antarctic eelpout-Q2** CCCAAACTAATGGGAATGCAAGTGAACAGGGCAGTGCCGTTGGGCACAACCCTCATGCCA

**Antarctic eelpout-Q3a** CCCAACCTAGTGGGAATGCAAGTGAACAGGGCAGTGCCGTTGGGCACAACCCTCATGCCA

**Antarctic eelpout-Q4** CCCAAACTAGTGGGAATGCAAGTGAACAGGGCAGTGCCGTTGGGCACAACCCTCATGCCA

**Antarctic eelpout-Q3b** TCCAAACTAGTGGGAATGCAAGTGAACAGGGCAGTGCCGTTGGGCACAACCCTCATGCCA

***P. brachycephalum*-Q2** CCCAGACTAGTGGGAATGCAAGTGAACAGGGCAGTGCCGTTGGGCACAACCCTCATGCCA

**Antarctic eelpout-Q5b** CCCAAACTAGTGGGAATGCAAGTGAACAGGGCAGTGTATCTGGACCAAACCCTCATGCCA

**Antarctic eelpout-Q6** CCCAAACTAGTGGGAATGCAAGTGAACAGGGCAGTGTATCTGGACGAAACCCTCATGCCA

***P. brachycephalum*-Q4** TCCAAAATAGTGGGAATGCAAGTGAACAGGGCAGTGAATCTGGACGAAACCCTCATGCCA

**ocean pout-Q6** CCCCAAATAGTCGGATTGCAAGTGGACAGGGCAGTGCCGTTGGGCACAACCCTCATGCCA

**ocean pout-Q7** TCCCAAATCGTGGGAAAGCAAGTGAACAGGGCAGTGCCGTTGGGCACAACCATCATGCCA

**Antarctic eelpout-sasB**  TGCAAAATGGTGGGAAAGACCGTGACGGAGGACGTGGAGGAGGACGACAGCGTCATGCCA

**wolf eel-sasB** TGCAAAATGGTGGGAAAGACCGTGACGGAGGATGTGGAGGAGGATGACAGCATCATGCCA

**Antarctic eelpout-sasA** TTCCAAATGGTGGGAAAGACCGTGACGAAGGACGTGGAGGAGGACGGCAGCCTCTTGCCA

**wolf eel-sasA** TTCCAAATGGTGGGAAAGACCGTGACGAAGGATGTGGAGGAGGACGACAGCCTCTTGCCA

**Atlantic wolffish-S1** TCCCAAATCGTGGGAAAGCAAGTGAACAGGCCAGTGGCTAAGGACGAAACCCTCATGCCA

**spotted wolffish-S1** TCCCAAATCGTGGGAAAGCAAGTGAACAGGCCAGTGGCTAAGGACGAAACCCTCATGCCA

**spotted wolffish-S2** TCCCAAATCGTGGGAAAGCAAGTGAACAGGCCAGTGGCTAAGGACGAAACCCTCATGCCA

**rock gunnel-S1** TCCAGAATAGTGGGAAAGCAAGTGAACCAGATAGTGGCTAAGGACCAAACCCTCATGCCA

**radiated shanny-S1** TCCACACTAGTGGGAAAGCAAGTGAACCAGCCAGTGGTTAAGGGCCAGACCCTCATGCGA

**ocean pout-S1** TCCCAAATAGTGGGGAAGCAAGTGAACACGCCAGTGGCTAAGGGCCAAACCCTCATGCCA

**ocean pout-S2** TCCCAAATAGTGGGGAAGCAAGTGAACAGGATAGTGGCTAAGGGCCAAACCCTCATGCCA

**ocean pout-S3** TCCCAAATGGTGGGGAAGCAAGTGAACAGGCCAGTGGCTAAGGGCCAAACCATCATGCCA

**ocean pout-S4** TCCCAAATAGTGGGGAAGCAAGTGAACACGCCAGTGGCTAAGGGCCAAACCATCATGCCA

**viviparous eelpout-S1** TCCCAAATAGTGGGGAAGCAAGTGAACACGCCAGTGGCTAAGGGCCAAACCCTCATGCCA

**notched-fin eelpout-S1** TCCCAAATAGTGGGGAAGCAAGTGAACACGCCAGTGGCTAAGGGCCAAACCCTCATGCCA

**viviparous eelpout-S2** TCCCAAATAGTGGGGAAGCAAGTGAACACGCCAGTGGCTAAGGGCCAAACCCTCATGCCA

**viviparous eelpout-S3** CTCCAAATAGTGGGGAAGCAAGTGAACGTGATAGTGGCTAAGGGCCAAACCATCATGCCA

**viviparous eelpout-S4** TCCCAAATAGTGGGGAAGCAAGTGAACGTGATAGTGGCTAAGGGCCAAACCATCATGCCA

**viviparous eelpout-S5** TCCCAAATAGTGGGGAAGCAAGTGAACGTGATAGTGCCTAAGGGCCATACCATCATGCCA

**notched-fin eelpout-S2** TCCCAAATAGTGGGGAAGCAAGTGAACGTGATAGTGGCTAAGGGCCAAACCCTCATGCCA

**viviparous eelpout-S6** TCCCAAATAGTGGGGAAGCAAGTGAACGTGATAGTGGCTAAGGGCCAAACCCTCATGCCA

**viviparous eelpout-S7** GACCAAATAGTGGGGAAGCAAGTGAACGTGATAGTGGCTAAGGGCCAAACCATCATGCCA

**viviparous eelpout-S8** GACCAAATAGTGGGGAAGCAAGTGAACGTGATAGTGGCTAAGGGCCAAACCATCATGCCA

**viviparous eelpout-S9** TCCCAAATAGTGGGGAAGCAAGTGAACGTGATAGTGGCTAAGGGCCAAACCATCATGCCA

**viviparous eelpout-S10** TCCCAAATAGTGGGGAAGCAAGTGAACGTGATAGTGCCTAAGGGCCAAACCATCATGCCA

**notched-fin eelpout-S3** TCCCAAATAGTGGGGAAGCAAGTGAACGTGATAGTGGCTAAGGGCCAAACCATCATGCCA

**notched-fin eelpout-S4** TCCCAAATAGTGGGGAAGCAAGTGAACGTGATAGTGGCTAAGGGCCAAACCCTCATGCCA

**notched-fin eelpout-Q1** D M V K F Y C L C A P K N *

**notched-fin eelpout-Q1** GACATGGTGAAATTTTACTGCCTCTGCGCGCCGAAG---AACTGA---------------

**viviparous eelpout-Q1** GACATGGTGAAATTTTACTGCCTCTGCGCGCCGAAG---AACTGAagatgccaaggagtt

**ocean pout-Q1** GACATGGTGAAATTTTACTGCCTCTGCGCGCCGAAG---AACTGAaggtgccaaggagtt

**ocean pout-2** GACATGGTGAAATTTTACTGCCTCTGCGCGCCGAAG---AACTGAaggtgccaaggagtt

**Atlantic wolffish-Q1** GACATGGTGAAATTTTACTGCCTCTGCGCGCCGAAG+32tcctgagggtgccaaggagtt

**Atlantic wolffish-Q2** GACATGGTGAAATTTTACTGCCTCTGCGCGCCGAAG---TACTGAaggtgccaaggagtt

**Alaskan ronquil-Q1**  GACATGGTGAAATTTTACTGCCTCTGCTGAaagaagtagttctgagggtgccaaggagtt

**ocean pout-Q3**  GACATGGTGAAAGGCTACCCCCTG---ACTTAGtag---tt-------------------

**notched-fin eelpout-Q2** GACATGGTGAAAGGGTACGCCCCG---GCTTAG---------------------------

**viviparous eelpout-Q2** GACATGGTGAAAGGGTACGCCCCG---GCTTAGtag---ttctgagggtgccaaggagtt

**ocean pout-Q4**  GGCATGGTGAAAACGTACACTCCA---GCAAAGTAG---ttctgagggtgccaaggagtt

**viviparous eelpout-Q3** GGCATGGTGAAAACGTACACTCCA---GTGAAGTAG---ttctgagggtgccaaggagtt

**notched-fin eelpout-Q3** GGCATGGTGAAAACGTACACTCCA---GTGAAGTAG------------------------

**ocean pout-Q5**  GACATGGTGAAAGGGTACCCTCCG---GCTTAGtag---ttctgagggtgccaaggagtt

**radiated shanny-Q1** GAGATGGTGAAAGGGTACAGCCCG---GCTAAGTAG---ttctgagggtgccaaggagtt

**rock gunnel-Q1** GACATGGTGAAAACGTACCAACCA---GCGAAGTAA---ttctgagggtgccaaggagtt

**viviparous eelpout-Q4** GACATGGTGAAAGGGTACACCCCG---GCTTAGtag---ttctgagggtgccaaggagtt

**notched-fin eelpout-Q4** GAGATGGTGAAAGGGTACACCCCG---GCTTAG---------------------------

**viviparous eelpout-Q5** GACATGGTGAAAGGGTACGCCCCG---AATTAGtag---ttctgaaggtgccaaggagtt

**spotted wolffish-Q1** GACATGGTGAAAGGGTACCCCCCG---AATTAGtag---ttctgagcgtgccaaggagtt

**spotted wolffish-Q2** GACATGGTGAAAGGGTACCCCCCG---AATTAGaag---tt-------------------

***P. brachycephalum*-Q1** GACATGGTGAAAATGTACTGCTTGTG*TATATAA------aatatacattcattcaatgtt*

**Antarctic eelpout-Q1** GACATGGTGAAAAACTAT---------GAGAAGTAG---ttctgagcgtgccaaggagtt

**Antarctic eelpout-Q2** GACATGGTGAAAAACTAT---------GAGAAGTAG---tt-------------------

**Antarctic eelpout-Q3a** GACATGGTGAAAAACTAT---------GAGGATGGG------------------------

**Antarctic eelpout-Q4** GATATGGTGAAAAACTAT---------GAGAAGTAG---ttctgagcgtgccaaggagtt

**Antarctic eelpout-Q3b** GATATGGTGAAAAACTAT---------GAGAAGTAG---ttctgagcgtgccaaggagtt

***P. brachycephalum*-Q2** GATATGGTGAAAAACTAT---------CAGAAGTAG---ttctgagcgtgccaaggagtt

**Antarctic eelpout-Q5b** GATATGGTGAAAAACTAT---------GAGGATGTG------------------------

**Antarctic eelpout-Q6** GATATGGTGAAAAACTAT---------GAGAAGTAG---ttctgagcgtgccaaggagtt

***P. brachycephalum*-Q4** GATATGGTGAAAACGTAT---------CAGAAGTAG---ttctgagcgtgccaaggagtt

**ocean pout-Q6** GACATGGTGAAAACGTACTGCGCG---GCGAAGTAG---ttctgagggtggcaaggagtt

**ocean pout-Q7** GAGATGGTGAAAGGGTACGCCCCG---AATTAGtag---tt-------------------

**Antarctic eelpout-sasB** GAGATGGTGAAAGGGTACTGCAAG---AACAAGAAG---TGCTGAggatggcaaggagtt

**wolf eel-sasB** GAGATGGTGAAAGGGTACTGCAAG---AACAAGAAG---TGCTGAggatggcaaggagtt

**Antarctic eelpout-sasA** GAGGTGGTGGACGGGTACTGCAAG---AAGAGGAAG---TGCTGAgggtcgcaaggagtt

**wolf eel-sasA** GAGGTGGTGGATGGGTACTGCAAG---AAGAGGAAG---TGCTGAggatggcaaggagtt

**Atlantic wolffish-S1** AACATGGTGAAAACGTACCGCGCG---GCAAAGTAG---ttctgagggtgccaaggagtt

**spotted wolffish-S1** AACATGGTGAAAACGTACCGCGCG---GCAAAGTAG---ttctgagggtgccaaggagtt

**spotted wolffish-S2** AACATGGTGAAAACGTACCGCGCG---GCAAAGTAG---ttctgagggtgccaaggagtt

**rock gunnel-S1** AGCATGGTGAAAACGTACCAACCA---GCGAAGTAG---ttctgagggtgccaaggagtt

**radiated shanny-S1** AATATGGTGAAACCATAGgca------aagaagttg---ttttgagggtgccaaggagtt

**ocean pout-S1** AACATGGTGAAAACGTACGTCGCG---GGAAAGTAG---ttctgagggtgccaaggagct

**ocean pout-S2** AACATGGTGAAAACGTACGCCGCG---GGAAAGTAG---ttctgagggtgccaaggggct

**ocean pout-S3** AACATGGTGAAAACGTACGCCGCG---GGAAAGTAG---ttctgagggtgccaaggagct

**ocean pout-S4** AACATGGTGAAAACGTACGCCGCG---GGAAAGTAG---ttctgagggtgccaaggagct

**viviparous eelpout-S1** GACATGGTGAAAACGTACGTCCCG---GCAAAGTAG------------------------

**notched-fin eelpout-S1** GGCATGGTGAAAACGTACGTCCCG---GCAAAGTAG------------------------

**viviparous eelpout-S2** GACATGGTGAAAACGTACGTCCCG---GCAAAGTAG------------------------

**viviparous eelpout-S3** AACATGGTGAAAACGTACGCCGCG---GGAAAGTAG---ttctgagggtgccaaggagct

**viviparous eelpout-S4** ATCATGGTGAAAACGTACGTCCCG---GCAAAGTAG---ttctgagggtgccaaggagct

**viviparous eelpout-S5** ATCATGGTGAAAACGTACGCG------GGAAAGTAG---ttctgagggtgccaaggagct

**notched-fin eelpout-S2** GACATGGTGAAAACGTACGCG------GGAAAGTAG------------------------

**viviparous eelpout-S6** GACATGGTGAAAACGTACGCG------GGAAAGTAG---ttctgagggtgccaaggagct

**viviparous eelpout-S7** GGCATGGTGAAAACGTACGCG------GGAAAGTAG---ttctgagggtgccaaggagct

**viviparous eelpout-S8** GACATGGTGAAAACGTACGCG------GGAAAGTAG---ttctgagggtgccaaggagct

**viviparous eelpout-S9** GACATGGTGAAAACGTACGCG------GGAAAGTAG---ttctgagggtgccaaggagct

**viviparous eelpout-S10** GACATGGTGAAAACGTACGCG------GGAAAGTAG---ttctgagggtgccaaggagct

**notched-fin eelpout-S3** ATCATGGTGAAAACGTACGCG------GGAAAGTAG------------------------

**notched-fin eelpout-S4** GACATGGTGAAAACGTACGCG------GGAAAGTAG------------------------
