# Supplementary figures and images for: Antifreeze protein dispersion in eelpouts and related fishes reveals migration and climate alteration within the last 20 Ma
Source: PLoS One. 2020 Dec 15;15(12):e0243273. doi: 10.1371/journal.pone.0243273 (PMC7737890; doi:10.1371/journal.pone.0243273)

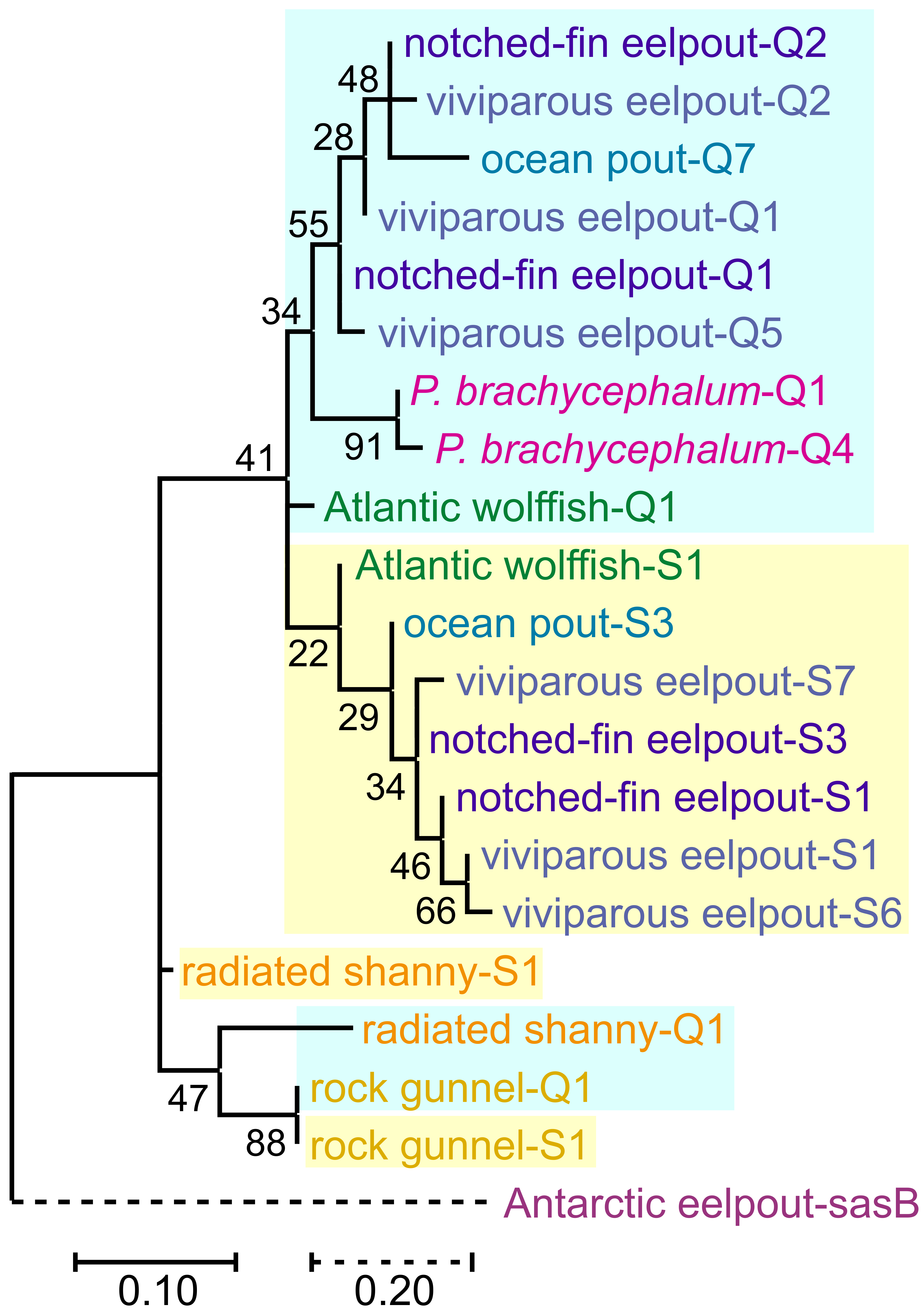

Supplement: S5 Fig — Cyan and yellow backing denotes QAE and SP isoforms, respectively, except for radiated shanny and rock gunnel sequences that cluster together on a separate branch. Bootstrap values (percent) are indicated at most nodes and the scale bar represents an average of 0.1 changes per site. Sequences are named as in Fig 2. (TIF) [file pone.0243273.s005.tif]

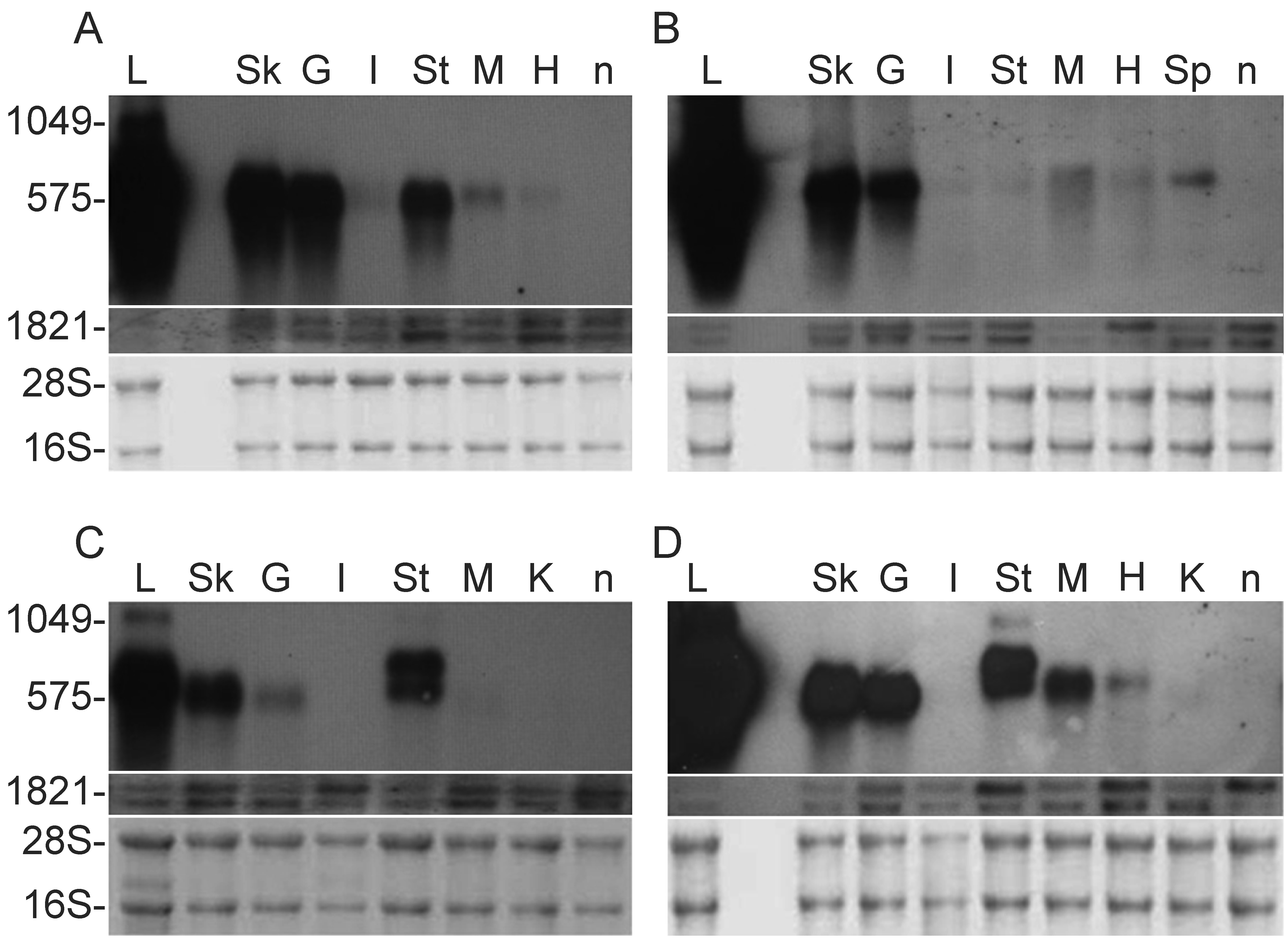

Supplement: S6 Fig — Northern blot analysis of total RNA from two rock gunnel individuals (A and B) and two radiated shanny individuals (C and D). The panels in each set show the hybridization signal to the AFP probe (top); the chicken β-tubulin probe (middle) cDNAs; or ethidium bromide staining of the 28S and 18S rRNA bands (bottom). The tissues are indicated as follows; L = liver, Sk = skin, G = gill, I = intestine, St = stomach, M = muscle, H = heart, Sp = spleen, K = kidney. RNA size marker positions are indicated on the left (bases) and total RNA from cunner skin was used as a negative control (n). (TIF) [file pone.0243273.s006.tif]

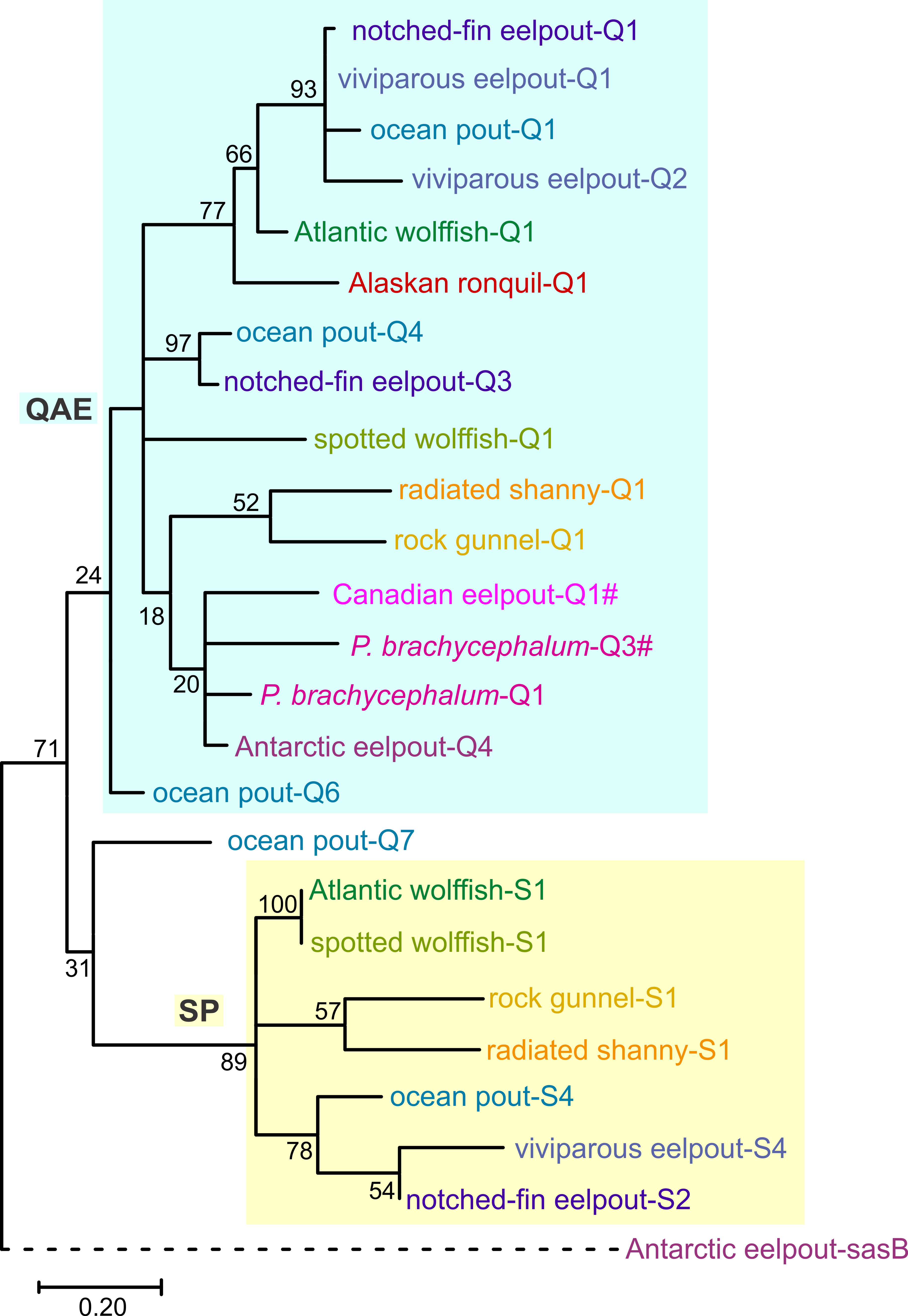

Supplement: S7 Fig — The amino acid sequences shown in Fig 2, along with two sequences determined solely by Edman degradation of purified proteins (Canadian eelpout-Q1 and P. brachycephalum-Q3, labelled with #) were used to generate a phylogenetic tree equivalent to Fig 3. (TIF) [file pone.0243273.s007.tif]

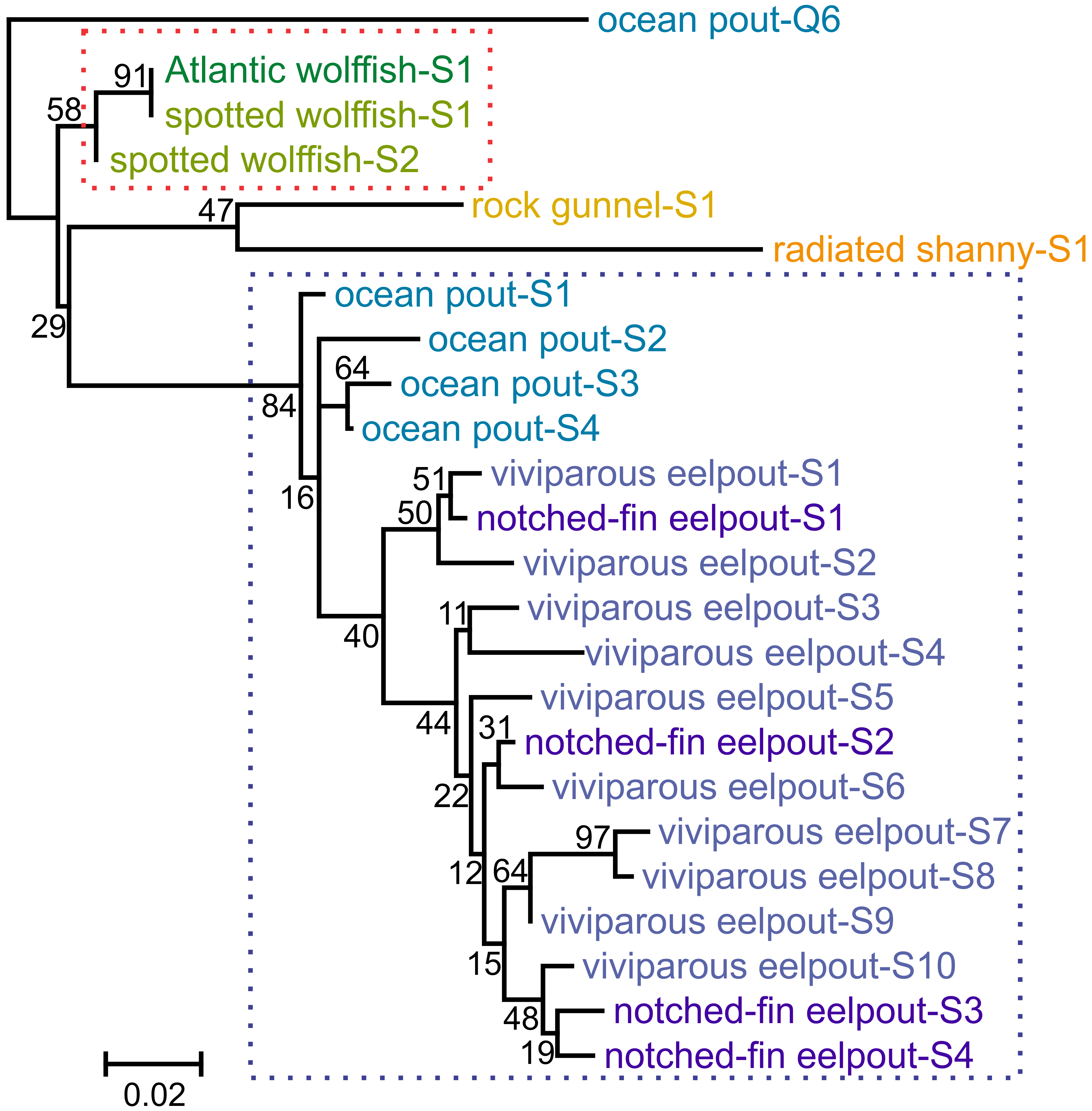

Supplement: S8 Fig — The nucleotide sequences of the SP subset of type III AFP sequences (S4 Fig) were used to generate a maximum-likelihood phylogenetic tree using a divergent isoform (ocean pout-Q6) as the outgroup. Bootstrap values (percent) are indicated at most nodes and the scale bar represents an average of 0.02 changes per site. Sequences are named as in Fig 2. (TIF) [file pone.0243273.s008.tif]

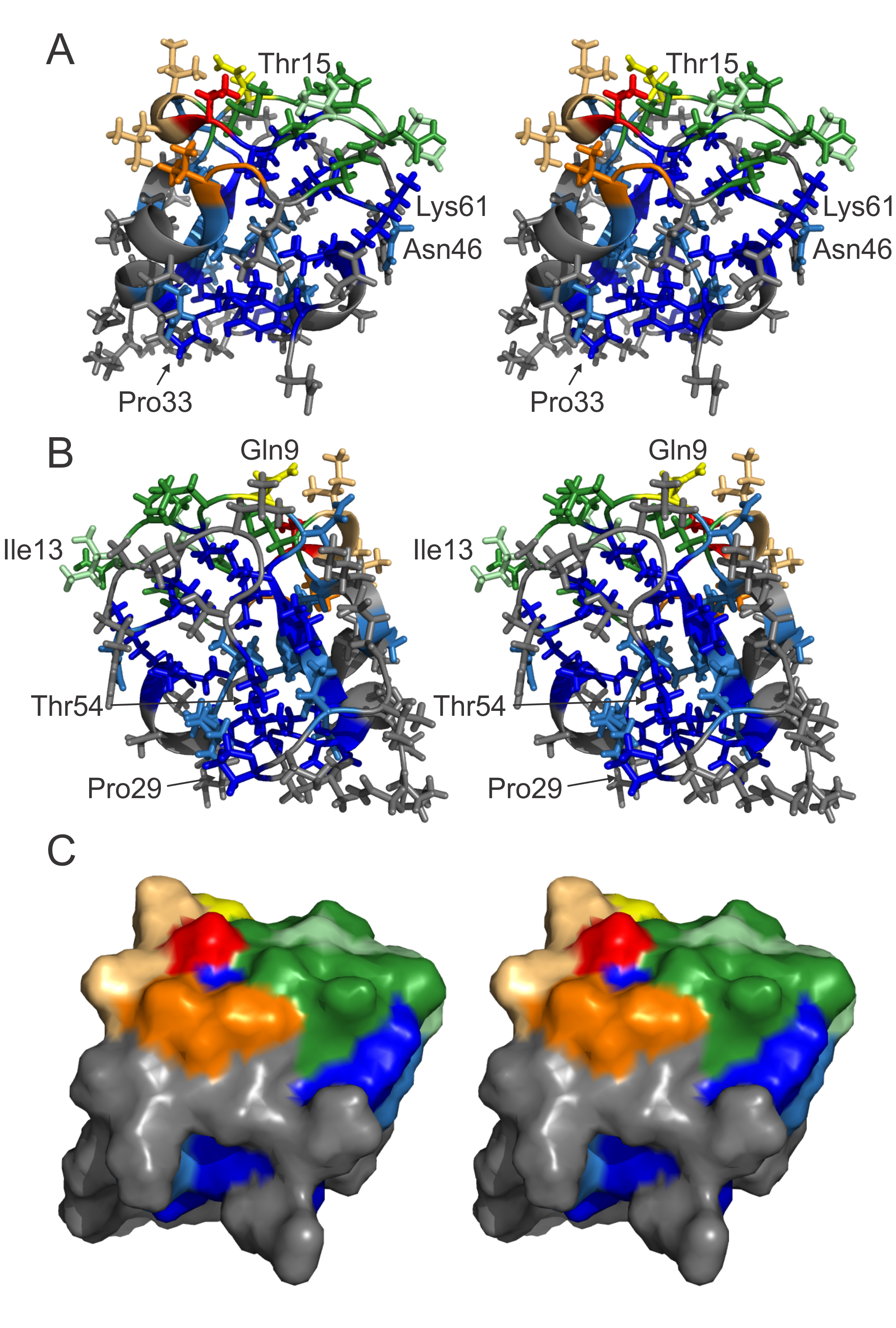

Supplement: S9 Fig — Residues that are absolutely, moderately or poorly conserved are colored as follows: respectively; pyramidal ice-binding plane, dark green, light green, yellow; prism ice-binding plane, red, orange, pale orange; rest of protein, dark blue, light blue, grey. Front view (A), back view (B) and front surface view (C). (TIF) [file pone.0243273.s009.tif]

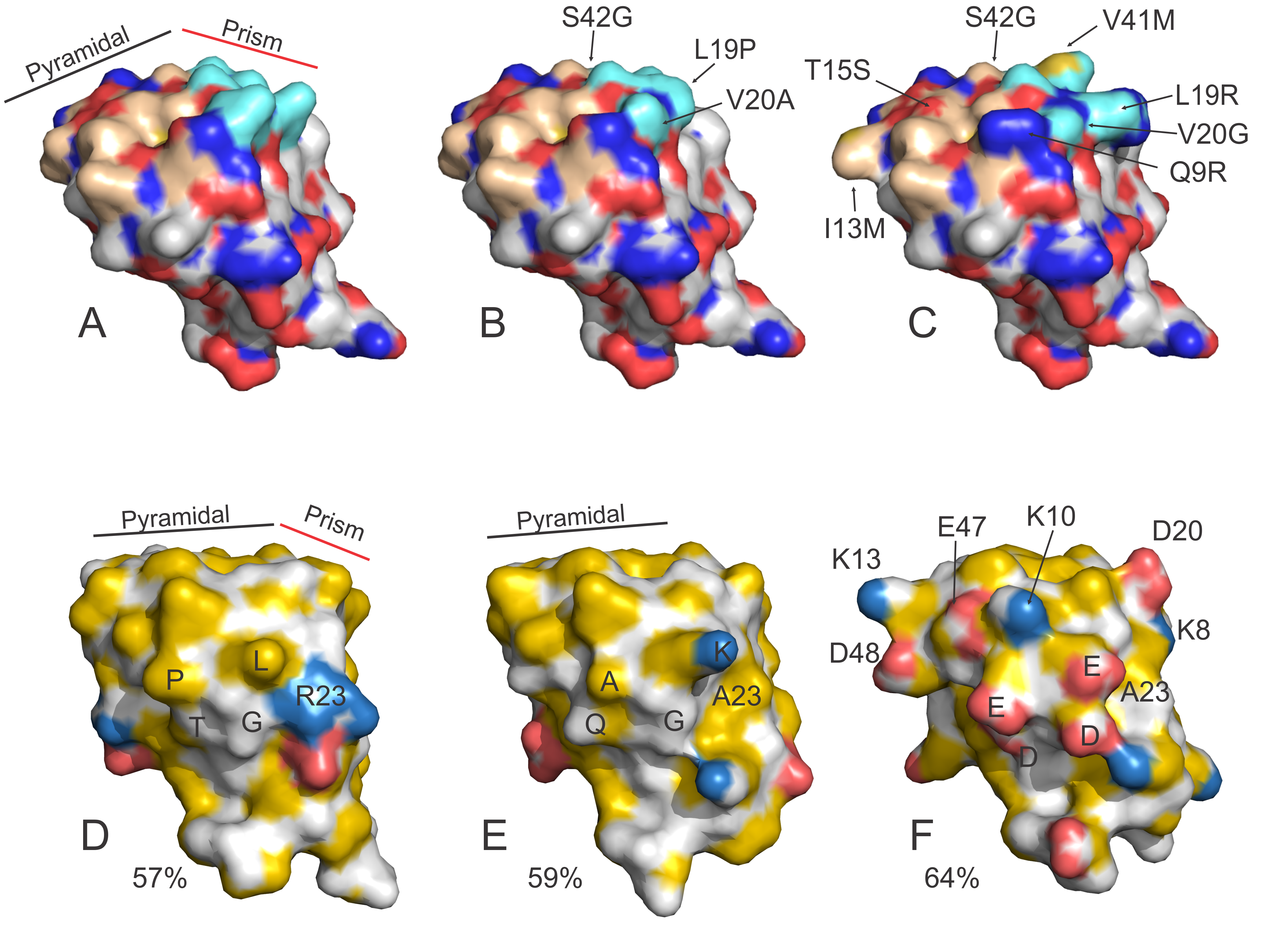

Supplement: S10 Fig — A) Wild-type QAE isoform, ocean pout-Q5 (HPLC12, PDB 1HG7) B) Ocean pout-Q5 with the introduction of the three ice-binding mutations found in P. brachycephalum-Q4 and C) A compilation of the most severe mutations at variable ice-binding residues (S3 Fig) introduced to ocean pout-Q5. Nitrogen is blue, oxygen is red, sulfur is yellow and carbon is pale orange (on the pyramidal-plane ice-binding surface), cyan (on the prism-plane ice-binding surface) or white (elsewhere). D) QAE isoform (PDB 4UR4) E) SP isoform (PDB 4UR6) F) Antarctic eelpout SAS-B residues mapped onto 4UR4 (excluding the last four residues of SAS-B). Atoms are colored by charge and hydrophobicity with red for charged oxygen, blue for charged nitrogen and yellow for carbon not bonded to nitrogen or oxygen. All other backbone and polar groups are colored white. Residues are numbered according to Fig 2, except residues 50–53 (PLGT, AKGQ and EEDD respectively). The percentage of the surface that is solvent accessible is indicated. (TIF) [file pone.0243273.s010.tif]
