# Supplementary material for: Antifreeze protein dispersion in eelpouts and related fishes reveals migration and climate alteration within the last 20 Ma
Source: PLoS One. 2020 Dec 15;15(12):e0243273. doi: 10.1371/journal.pone.0243273 (PMC7737890; doi:10.1371/journal.pone.0243273)
Supplement: S1 Table — *F is forward and R is reverse direction. (DOCX) [file pone.0243273.s011.docx]

| **^*^Primer Name** | **Nucleotide sequence (5ʹ-3ʹ)** |
| --- | --- |
| AR_F2 | TTCGTCCTCCTTTGTGTCGAC |
| AR_R1 | CACAGGTTTTGACATGTTCAGTTA |
| AR_F3 | CTGATCCCCATNAATACTGCC |
| AR_R2 | TGGGAAGAAACTCCTTGGCACC |
| OPRT_F | CGGTTTGCTTTTCGTCCTCCT |
| S&G_R | GGAAGAAACTCCTTGGCACCCTCAGA |
| QAE_R | CAGACTGACTAGTCGGGGAATGTCCT |
| SP_R | CTGGYCCTTAGCCACTRKCTGGTTCAC |
| S&G_F | CACTTCATTCTCCGCTAATTAATTAATT |
| Probe_F | 5′-GTGCCAAGGAGTTTCTTCCCAA -3′ |
| Probe_R | 5′-CCATCAAATCTCAACATAGTCTCC-3′ |
