# Supplementary material for: Antifreeze protein dispersion in eelpouts and related fishes reveals migration and climate alteration within the last 20 Ma
Source: PLoS One. 2020 Dec 15;15(12):e0243273. doi: 10.1371/journal.pone.0243273 (PMC7737890; doi:10.1371/journal.pone.0243273)
Supplement: S2 Table — 1Excludes poly(A) tail. 2Includes STOP codon. 3Excludes STOP codon and poly(A) tail. 4Presumes cleavage of C-terminal Lys. (DOCX) [file pone.0243273.s012.docx]

|  | **Feature** | **rock gunnel-Q1** | **rock gunnel-S1** | **radiated shanny-Q1** | **radiated shanny-S1** |
| --- | --- | --- | --- | --- | --- |
| cDNA | GenBank accession # | JX178758 | JX178757 | JX178756 | JX178755 |
|  | ^1^Total length (bp) | 534 | 528 | 534 | 522 |
|  | 5`UTR (bp) | 77 | 77 | 77 | 77 |
|  | ^2^ORF (bp) | 273 | 267 | 273 | 249 |
|  | ^3^3`UTR (bp) | 184 | 184 | 184 | 196 |
|  | STOP codon | TAA | TAG | TAG | TAG |
|  | Polyadenylation signal | AATAAA | AATAAA | AATAAA | AATAAA |
| Protein | GenBank accession # | AFQ32241 | AFQ32240 | AFQ32239 | AFQ32238 |
|  | # a.a. encoded | 90 | 88 | 90 | 82 |
|  | ^4^Mature protein length | 67 | 65 | 67 | 61 |
|  | Molecular mass (kDa) | 7.15 | 6.82 | 6.91 | 6.51 |
|  | Isoelectric point | 9.52 | 9.82 | 6.30 | 10.17 |
