# Supplementary material for: Antifreeze protein dispersion in eelpouts and related fishes reveals migration and climate alteration within the last 20 Ma
Source: PLoS One. 2020 Dec 15;15(12):e0243273. doi: 10.1371/journal.pone.0243273 (PMC7737890; doi:10.1371/journal.pone.0243273)
Supplement: S3 Table — SP and QAE isoforms are highlighted yellow and cyan, respectively, with the QAE sequences that diverged early are highlighted grey. Protein accession numbers are used for consistency between figures. Percent identity excludes gaps, and sequences known only from Edman degradation are underlined. Sequences denoted with an asterisk were not included in the alignments as they differed at two or fewer a.a. resides from isoforms that were included. (DOCX) [file pone.0243273.s013.docx]

| Sequence and source | | | | Closest Conspecific Nucleotide Match | | | | Closest Conspecific Protein Match | | |
| --- | --- | --- | --- | --- | --- | --- | --- | --- | --- | --- |
| This study^1^ | | Source | Code | Accession | Source | Code | Identity (%) (bp in gaps) | Accession | Source | Identity (%) |
| ALL26673 | Blood | | ocean pout-Q4 | ALL26679 | muscle | ocean pout-Q6 | 93 (0) | ALL26675 | skin | 88 |
| ALL26674 | Gill | | ocean pout-Q1 | AAA49348 | genomic | ocean pout-Q2 | 97 (0) | P19606 | genomic | 90 |
| ALL26675 | Skin | | ocean pout-Q5 | ALL26673 | blood | ocean pout-Q4 | 94 (28) | P19614 HPLC12 | serum | 100 |
| ALL26676 | Liver | | ocean pout-S4 | AAA49347 | liver | ocean pout-S1 | 98 (0) | P19608 HPLC1 | serum | 100 |
| ALL26677 | Liver | | ocean pout-S2 | ALL26678* | liver | ocean pout-Q1 | 97 (0) | P19611 HPLC7 | serum | 100 |
| ALL26678* | Gill | | OP5* | AAA49346 | genomic | OP5* | 100 (0) | P19607 OP5* | genomic | 100 |
| ALL26679 | Muscle | | ocean pout-Q6 | ALL26673 | blood | ocean pout-Q4 | 93 (0) | ALL26673 | blood | 81 |
| ALL26680* | Muscle | | ocean pout-Q7 | ABA41372 | pancreas | ocean pout-Q7 | 100 (0) | ABA41372 | pancreas | 100 |
