# Supplementary material for: Antifreeze protein dispersion in eelpouts and related fishes reveals migration and climate alteration within the last 20 Ma
Source: PLoS One. 2020 Dec 15;15(12):e0243273. doi: 10.1371/journal.pone.0243273 (PMC7737890; doi:10.1371/journal.pone.0243273)
Supplement: S4 Table — (DOCX) [file pone.0243273.s014.docx]

| **Isoform** | **Run** | **Read numbers** |
| --- | --- | --- |
| viviparous eelpout-Q1 | SRR013409 | 210424, 230210 |
| viviparous eelpout-Q2 | SRR013409 | 14909, 6667 |
| viviparous eelpout-Q3 | SRR013409 | 229078, 172240 |
| viviparous eelpout-Q4 | SRR013409  SRR013339 | 56060  16917 |
| viviparous eelpout-Q5 | SRR013409 | 95039, 53326 |
| viviparous eelpout-S3 | SRR013409 | 211678, 250361 |
| viviparous eelpout-S4 | SRR013409 | 111206, 200358 |
| viviparous eelpout-S5 | SRR013409 | 139939, 51323 |
| viviparous eelpout-S6 | SRR013409 | 88772, 109335 |
| viviparous eelpout-S7 | SRR013409, SRR013410 | 4636  67249 |
| viviparous eelpout-S8 | SRR013409 | 230402 |
| viviparous eelpout-S9 | SRR013409 | 29826, 184418 |
| viviparous eelpout-S10 | SRR013409 | 139067, 226551 |
| *P. brachycephalum*-Q1 | SRR405100 | 21616 |
| *P. brachycephalum*-Q2 | SRR405100 | 271136 |
