# Supplementary material for: Antifreeze protein dispersion in eelpouts and related fishes reveals migration and climate alteration within the last 20 Ma
Source: PLoS One. 2020 Dec 15;15(12):e0243273. doi: 10.1371/journal.pone.0243273 (PMC7737890; doi:10.1371/journal.pone.0243273)
Supplement: S5 Table — (DOCX) [file pone.0243273.s015.docx]

| **Isoform** | **Accession Number** | **Isoform** | **Accession Number** |
| --- | --- | --- | --- |
| ocean pout-Q1 | BAD95788 | ocean pout-Q6 | ALL26679 |
| notched-fin eelpout-Q1 | ALL26674 | Antarctic eelpout-Q5b | AFU88725 |
| ocean pout-Q2 | AAA49348 | Antarctic eelpout-Q6 | AFU88746 |
| Atlantic wolffish-Q1 | AFJ52154 | ocean pout-Q7 | ABA41372 |
| Atlantic wolffish-Q2 | AFJ52156 | Atlantic wolffish-S1 | AFJ52149 |
| Alaskan ronquil-Q1 | ALF40136 | spotted wolffish-S1 | AFJ52151 |
| ocean pout-Q3 | ABA41371 | spotted-wolffish-S2 | AFJ52152 |
| notched-fin eelpout-Q2 | BAD95786 | rock gunnel-S1 | AFQ32240 |
| ocean pout-Q4 | ALL26673 | radiated shanny-S1 | AFQ32238 |
| notched-fin eelpout-Q3 | BAD95785 | ocean pout-S1 | AAA49347 |
| ocean pout-Q5 | ALL26675 | ocean pout-S2 | ALL26677 |
| radiated shanny-Q1 | AFQ32239 | ocean pout-S3 | P19605 |
| rock gunnel-Q1 | AFQ32241 | ocean pout-S4 | ALL26676 |
| notched-fin eelpout-Q4 | BAD95783 | viviparous eelpout-S1 | AGM97733 |
| Canadian eelpout-Q1 | P24028 | notched-fin eelpout-S1 | BAD95781 |
| spotted wolffish-Q1 | ABA41377 | viviparous eelpout-S2 | ABN42204 |
| spotted wolffish-Q1 | ABA41378 | notched-fin eelpout-S2 | BAD95780 |
| Antarctic eelpout-Q1 | AAB60609 | notched-fin eelpout-S3 | BAD95778 |
| Antarctic eelpout-Q2 | ABA41374 | notched-fin eelpout-S4 | BAD95777 |
| Antarctic eelpout-Q3a | ACF39777 | Antarctic eelpout-sasB | ADJ80992 |
| Antarctic eelpout-Q3b | ACF39777 | wolf-eel-sasB | XP_031720499 |
| Antarctic eelpout-Q4 | AFU88745 | wolf-eel sasA | XP_031720498 |
| *P. brachycephalum*-Q3 | P12101 | Antarctic eelpout-sasA | ADJ80990.1 |
| *P. brachycephalum*-Q4 | ABA41375 |  |  |
