# Supplementary material for: Antifreeze protein dispersion in eelpouts and related fishes reveals migration and climate alteration within the last 20 Ma
Source: PLoS One. 2020 Dec 15;15(12):e0243273. doi: 10.1371/journal.pone.0243273 (PMC7737890; doi:10.1371/journal.pone.0243273)
Supplement: S1 File — (DOCX) [file pone.0243273.s016.docx]

Article

Supplementary Information for

**Antifreeze protein dispersion in eelpouts and related fishes reveals migration and climate alteration within the last 20 Ma**

**Rod S. Hobbs^1^, Jennifer R. Hall^2^, Laurie A. Graham^3*^, Peter L. Davies^3^, Garth L. Fletcher^1^**

* Corresponding author: Laurie A. Graham. Email: [grahamla@queensu.ca](mailto:grahamla@queensu.ca)

**This PDF file includes:**

Supplementary materials and methods

Supplementary results

SI References

**Supplementary Materials and methods**

**Purification of DNA and RNA from tissue samples**

The Alaskan ronquil tissue sample had been stored in 95% ethanol, so the ethanol was removed by soaking the tissue in the following series of ethanol/phosphate buffered saline (PBS) washes for five min each: 75% ethanol/25% PBS; 50% ethanol/50% PBS; 25% ethanol/75% PBS; 100% PBS. DNA was then extracted using the DNeasy Blood and Tissue Kit (QIAGEN, Mississauga, ON) following the manufacturer’s instructions. DNA integrity was verified by 0.5% agarose gel electrophoresis and purity was assessed by A260/280 and A260/230 ratios.

Radiated shannys, rock gunnels and Atlantic Ocean pout were maintained in 250 L aquaria supplied with flowing seawater (32‰ to 33‰ salinity) at seasonally ambient water temperature and photoperiod [1]. Fish were euthanized via an MS-222 overdose and tissues were removed quickly. Tissues were immediately either snap frozen and stored at -70°C, or homogenized in TRIzol Reagent (Invitrogen/Life Technologies, Burlington, ON). Total RNA was extracted from frozen or fresh tissue using TRIzol Reagent following the manufacturer’s instructions. RNA integrity was verified by 1% agarose gel electrophoresis and purity was assessed by A260/280 and A260/230 ratios.

**Cloning of the type III AFP (QAE isoform) partial gene sequence from Alaskan ronquil**

The sequences of all primers used in gene cloning are presented in Table S4. Primers were designed based upon consensus sequences from conserved areas of aligned piscine type III AFP gene sequences. The partial gene sequence for type III AFP from Alaskan ronquil was amplified in two overlapping fragments using semi-nested PCR. PCR amplification (primary and semi-nested) was performed using DyNAzyme EXT DNA polymerase (Thermo Fisher Scientific, Ottawa, ON). Briefly, 50-μl reactions were prepared containing DyNAzyme EXT DNA polymerase (1 U), the manufacturer’s Optimized DyNAzyme EXT Buffer (1X final concentration), 0.2 mM dNTPs, 0.2 μM each of forward and of reverse primer, and template DNA. PCR cycling conditions were 40 cycles of [94°C for 30 sec, 65°C decreasing by 0.5°C per cycle (to 45.5°C at cycle 40) for 30 sec and 72°C for 1 min]. In the primary reaction, 10 ng of genomic DNA was used as template with primers AR_F2 and AR_R1. In the semi-nested reactions, one-fiftieth of the first reaction was used as template with primer pairs AR_F3/AR_R1 and AR_F2/AR_R2.

**Cloning of the type III AFP (QAE and SP isoforms) cDNAs from radiated shanny, rock gunnel and Atlantic Ocean pout**

The sequences of all primers used in cDNA cloning are presented in Table S4. Full length cDNAs for type III AFP from radiated shanny and rock gunnel were cloned using a commercial kit for RNA ligase-mediated rapid amplification of 5ʹ and 3ʹ cDNA ends (RLM-RACE) [GeneRacer Kit (Invitrogen/Life Technologies)]. Partial cDNAs for type III AFP from Atlantic Ocean pout were cloned using the 3ʹ RACE protocol only.

For 3ʹ RACE, a modification of the GeneRacer Kit protocol, in which the steps for ligation of the GeneRacer RNA oligonucleotide to full-length mRNAs were omitted, was used. Briefly, total RNA (1 μg) extracted from radiated shanny liver and skin, rock gunnel liver and skin, and Atlantic ocean pout blood, gill, liver, muscle and skin was reverse-transcribed in separate 20-μl reactions using the GeneRacer oligo-dT primer [500 ng (Invitrogen/Life Technologies)], dNTPs [0.5 mM final concentration (Invitrogen/Life Technologies)] and Superscript III reverse transcriptase [200 U (Invitrogen/Life Technologies)] with the manufacturer’s first strand buffer (1X final concentration) and DTT (10 mM final concentration) at 50°C for 1 h. Touchdown PCR amplification was then performed for each of the cDNA templates using Taq DNA polymerase (Invitrogen/Life Technologies), an Atlantic ocean pout type III AFP-specific 5' primer (OPRT_F) and the GeneRacer 3' primer. Briefly, 50-μl reactions were prepared containing Taq DNA polymerase (2 U), the manufacturer’s PCR buffer (1X final concentration), 1.5 mM MgCl_2,_ 0.2 mM dNTPs, 0.2 μM each of forward and of reverse primer, and 100 ng of cDNA (representing 100 ng of input total RNA). PCR cycling conditions were 13 cycles of [94°C for 45 sec, 67°C decreasing by 1°C per cycle (to 55°C at cycle 13) for 30 sec and 72°C for 30 sec] and then 19 cycles of [94°C for 45 sec, 55°C for 30 sec and 72°C for 30 sec].

For 5ʹ RACE, four separate 5ʹ RACE-ready cDNAs were constructed using total RNA extracted from radiated shanny liver and skin, and rock gunnel liver and skin following the manufacturer’s instructions. Nested touchdown PCR was then used to amplify the 5ʹ ends of the cDNAs. PCR core reaction component concentrations were as described for 3ʹ RACE. In the primary reaction, the cDNAs that had been reverse-transcribed from liver and skin were pooled for each species and used as the template with the GeneRacer 5' primer and the S&G_R primer. PCR cycling conditions were 11 cycles of [94°C for 30 sec, 72°C decreasing by 1°C per cycle (to 62°C at cycle 11) for 30 sec and 72°C for 1 min] and then 20 cycles of [94°C for 30 sec, 62°C for 30 sec and 72°C for 1 min]. In the nested reaction, the primary PCR products were diluted 20 x with water and used as template with the GeneRacer 5' nested primer and either the QAE_R or the SP_R primer. PCR cycling conditions were eight cycles of [94°C for 30 sec, 72°C decreasing by 1°C per cycle (to 65°C at cycle 11) for 30 sec and 72°C for 1.5 min] and then 25 cycles of [94°C for 30 sec, 65°C for 30 sec and 72°C for 1.5 min].

The full-length cDNA sequences for both type III AFPs from radiated shanny and rock gunnel were then verified using 3ʹ RACE with a sense primer located near the 5ʹ end of the cDNA. Touchdown PCR was performed with the GeneRacer 3' primer and the S&G_F primer. Template cDNAs, PCR core reaction components and cycling conditions were as described for nested 5ʹ RACE.

**PCR product sequencing**

All PCR products were sequenced using the following protocol. PCR products were electrophoresed on a 1% agarose gel, excised and purified using the QIAquick Gel Extraction Kit (QIAGEN). They were then subcloned into pGEM-T Easy (Thermo Fisher Scientific), and transformations performed using Subcloning Efficiency DH5α Competent Cells (Invitrogen/Life Technologies) and standard molecular biology techniques. Plasmid DNA was isolated from individual clones using the QIAprep Spin Miniprep Kit (QIAGEN) and clone restriction fragments screened for inserts by visual comparison with a DNA size marker (1 kb Plus DNA Ladder; Invitrogen/Life Technologies) using 1.0% agarose gel electrophoresis. Subclones were sequenced in both directions using BigDye Terminator reagents (Applied Biosystems/Life Technologies) and the 3730xl DNA Analyzer (Applied Biosystems/Life Technologies) at the Genomics and Proteomics Facility, CREAIT Network, Memorial University. Sequence data were extracted using Sequence Scanner v1.0 (Applied Biosystems/Life Technologies) and compiled and analyzed using Vector NTI and AlignX (Vector NTI Advance 11, Life Technologies).

**Northern blot analysis**

Formaldehyde agarose gel electrophoresis was performed using total RNA from a variety of tissues following the manufacturer’s instructions (DIG Northern Starter Kit, Roche Applied Science), with the following modifications. For each tissue, total RNA (2 μg) was prepared in a final volume of 5 μl. Fifteen μl of freshly prepared loading buffer [100% formamide (250 μl); 37% formaldehyde (83 μl); 10X MOPS (50 μl); 100% glycerol (50 μl); 2.5% bromophenol blue (10 μl); water (57 μl); ethidium bromide (6 μg)] was added to each sample, mixed and heated at 70 °C for 10 min and briefly chilled on ice. Total RNA was electrophoresed on a 1.2% formaldehyde agarose gel prepared with 1X MOPS buffer and 0.22 M formaldehyde at 130 V. Loading was evaluated by visualizing the ribosomal RNA bands using ethidium bromide prior to transfer to nylon membranes.

The antisense RNA probe was generated from a 140-bp segment within the 3ʹ UTR of the rock gunnel QAE cDNA that shares >96% identity with the isoforms obtained from both radiated shanny and rock gunnel. It was amplified using primers Probe_F and Probe_R (Table S4), from a plasmid containing the rock gunnel QAE 3ʹ RACE PCR product as template. PCR core reaction component concentrations and cycling conditions were as described for 3ʹ RACE. The amplicon was gel purified, subcloned and sequenced as described previously. Plasmid DNA containing the correct cDNA sequence (10 µg) was linearized with *Spe*I and then purified using the QIAquick Gel Extraction Kit (QIAGEN). The linearized plasmid was used to generate labeled antisense RNA in an *in vitro* transcription reaction with digoxigenin-11-UTP and T7 RNA polymerase following the manufacturer’s instructions (DIG Northern Starter Kit, Roche Applied Science, Laval, QC). A plasmid containing a 1111-bp beta-tubulin partial cDNA sequence from chicken (*Gallus gallus*) (GenBank acc. no.V00389) was used to create the DIG-labeled internal control probe using the same protocol.

RNA was transferred to a positively charged nylon membrane (Roche Applied Science) using a vacuum blotter (VacuGene XL Vacuum Blotting System, Amersham Biosciences, Piscataway, NJ) with 10X SSC buffer, and UV crosslinked (UV Stratalinker, Stratagene, La Jolla, CA). Pre-hybridization, hybridization and immunological detection were performed following the manufacturer’s instructions (DIG Northern Starter Kit, Roche Applied Science). The bands were visualized by exposure to BioMax Light chemiluminescent film (Kodak, Rochester, NY) for 30-60 min and processed with GBX Developer/Fixer (Kodak) following the manufacturer’s instructions. Films were scanned and analyzed using the AlphaImager 1220 Documentation and Analysis System (Alpha Innotech Corporation, San Leandro, CA).

**Protein structure analysis**

Protein structures were downloaded from the protein database (PDB) and rendered using PyMOL 1.7.6.3. Waters and hydrogens are not shown, and stereoscopic images were generated in parallel viewing mode rather than cross-eyed mode. Known mutations in ice-binding residues were introduced using the mutagenesis wizard.

**Supplementary Results**

**Type III AFP is most strongly expressed in the liver of rock gunnel and radiated shanny**

The tissue expression profile of type III AFP transcripts was assessed in two individuals (A, B) from rock gunnel and from radiated shanny (C, D) using northern blot analysis (Fig S7). As the probe sequence is not isoform-specific, this analysis provides a general overview of type III AFP transcript levels in different tissues. Hybridization of the AFP probe to the RNA from the skin of the cunner (*Tautogolabrus adspersus*), a fish that does not produce type III AFP, was not detected on any blot. Furthermore, a single hybridization signal was detected at approximately 600 bp in most positive tissues; consistent with the length of the sequences cloned herein (Table S3).

In rock gunnel, type III AFP transcripts were most abundant in liver and were moderately expressed in skin and gill. Moderate levels were found in the stomach from one individual (Fig S7A), but not the other (Fig S7B), even though the controls showed comparable levels of mRNA in both samples. Levels were low but detectable in muscle, heart and intestine in both individuals and in the spleen of fish B (the spleen of fish A was not tested).

In radiated shanny, type III AFP levels were also most abundant in liver and moderate in the skin and stomach of both individuals (Fig S7C, D). However, the levels in the gill and muscle of fish D were much higher than in fish C. Some signal was detected in heart (only tested in fish D) but signal was not detected in the kidneys or intestines of either fish. One interesting anomaly is that the sizes of the transcripts in stomach differ from the sizes in other tissues.

**Rock gunnel and radiated shanny AFP expression is similar to that found in other zoarcids**

Tissue-specific mRNA expression patterns observed in this study are similar to those seen in other Zoarcales family members including ocean pout [2,3], and Atlantic and spotted wolffish [4]. In all these studies, expression of type III AFP mRNA has been observed in multiple body tissues, with high levels noted in the liver. The liver was also noted as a major site of synthesis in the Antarctic zoarcids [5]. This is consistent with the liver typically functioning as the main secretory organ for plasma proteins in fish [6]. The differences in tissue expression between individuals may be a reflection of how variable the gene copy number can be between individuals of the same species. Tissue-specific expression differences were observed in the wolffish [4] along with gene copy differences in both ocean pout and wolffish [2,4]. The unique sizes of the transcripts from the stomach of the radiated shanny, as well as the multiple intense bands observed in Southern blots of both shanny and rock gunnel genomic DNA [7], suggests that both species possess many isoforms, some of which are likely tissue specific.

**References**

1. Fletcher GL. Circannual cycles of blood plasma freezing point and Na+ and Ci- concentrations in Newfoundland winter flounder (Pseudopleuronectes americanus): correlation with water temperature and photoperiod. Can J Zool. 1977;55: 789–795.

2. Hew CL, Wang NC, Joshi S, Fletcher GL, Scott GK, Hayes PH, et al. Multiple genes provide the basis for antifreeze protein diversity and dosage in the ocean pout, Macrozoarces americanus. J Biol Chem. 1988;263: 12049–12055. Available: http://www.ncbi.nlm.nih.gov/pubmed/3403560

3. Hobbs RS, Fletcher GL. Tissue specific expression of antifreeze protein and growth hormone transgenes driven by the ocean pout (Macrozoarces americanus) antifreeze protein OP5a gene promoter in Atlantic salmon (Salmo salar). Transgenic Res. 2008;17: 33–45. doi:10.1007/s11248-007-9128-5

4. Desjardins M, Graham LA, Davies PL, Fletcher GL. Antifreeze protein gene amplification facilitated niche exploitation and speciation in wolffish. FEBS J. 2012;279: 2215–2230. doi:10.1111/j.1742-4658.2012.08605.x

5. Cheng C-HC, Cziko PA, Evans CW. Nonhepatic origin of notothenioid antifreeze reveals pancreatic synthesis as common mechanism in polar fish freezing avoidance. Proc Natl Acad Sci. 2006;103: 10491–10496. doi:10.1073/pnas.0603796103

6. Fletcher GL, Hew CL, Davies PL. Antifreeze proteins of teleost fishes. Annu Rev Physiol. 2001;63: 359–90. doi:10.1146/annurev.physiol.63.1.359

7. Shears M, Kao MH, Scott GK, Davies PL, Fletcher GL. Distribution of type III antifreeze proteins in the Zoarcoidei. Mol Mar Biol Biotechnol. 1993;2: 104–111.
