# Supplementary material for: Antifreeze protein dispersion in eelpouts and related fishes reveals migration and climate alteration within the last 20 Ma
Source: PLoS One. 2020 Dec 15;15(12):e0243273. doi: 10.1371/journal.pone.0243273 (PMC7737890; doi:10.1371/journal.pone.0243273)
Supplement: S1 Raw image — (PDF) [file pone.0243273.s017.pdf]

```
If there are any blot/gel figures in your manuscript or its Supporting
Information for which you cannot provide the original uncropped and
unadjusted images please specify this in your response and provide details as
to which figures are affected by raw image data unavailability.
```

We have one figure (Fig. S6) in the Supporting Information section that is a composite of four northern (RNA) blots. This is the only blot/gel figure in the manuscript. Unfortunately, we do not have the original files for this figure because they were left on a gel doc system that has since been discarded at the end of its useful life. I can vouch that that the images have not been differentially enhanced or altered. They are not misrepresenting any aspect of the results. Each of the four panels in Fig S6 is in three sections showing an autoradiograph of the blot probed with an antifreeze sequence, re-probed with a tubulin sequence, placed above a photograph of the ethidium bromide-stained gel prior to blotting. The latter two parts of the images serve as loading controls for the blots. The data in Fig. S6 are not central to the main discoveries of our paper, but they do add useful information about the tissues in which antifreeze proteins are expressed in some species. We are appealing to leave this figure in the manuscript in the absence of the raw data files.
